# Protective effect of hepatocyte-enriched lncRNA-*Mir122hg* by promoting hepatocyte proliferation in acute liver injury

**DOI:** 10.1038/s12276-022-00881-2

**Published:** 2022-11-24

**Authors:** Zhenjun Yu, Yuhan Li, Shuai Shao, Beichen Guo, Mengxia Zhang, Lina Zheng, Kun Zhang, Feng Zhou, Li Zhang, Chiyi Chen, Wentao Jiang, Wei Hong, Tao Han

**Affiliations:** 1 Department of Hepatology and Gastroenterology, The Third Central Clinical College of Tianjin Medical University; Department of Histology and Embryology, School of Basic Medical Sciences, Tianjin Medical University, Tianjin, China; 2 Department of Gastroenterology and Hepatology, Tianjin Union Medical Center, Tianjin Medical University, Tianjin, China; 3 Department of Histology and Embryology, School of Basic Medical Sciences, Tianjin Medical University, Tianjin, China; 4 Liver transplant department, Organ transplant center, Tianjin First Center Hospital, Tianjin, China

**Keywords:** Long non-coding RNAs, Cell growth

## Abstract

Some long noncoding RNAs (lncRNAs), which harbor microRNAs in their gene sequence and are also known as microRNA host gene derived lncRNAs (lnc-MIRHGs), play a dominant role alongside miRNAs, or both perform biological functions synergistically or independently. However, only a small number of lnc-MIRHGs have been identified. Here, multiple liver injury datasets were analyzed to screen and identify the target lncRNA *Mir122hg*. *Mir122hg* was mainly enriched in liver tissues with human-mouse homology. In both CCl_4_-induced acute liver injury and Dgal/LPS-induced fulminant liver failure in mice, *Mir122hg* was sharply downregulated at the early stage, while a subsequent significant increase was only found in the CCl_4_ group with liver recovery. Overexpression and silencing assays confirmed that *Mir122hg* played a protective role in acute injury by promoting hepatocyte proliferation in vivo and in vitro. Consistent with the results of gene enrichment analysis, *Mir122hg* binding to C/EBPα affected its transcriptional repression, promoted gene transcription of downstream chemokines, *Cxcl2*, *Cxcl3*, and *Cxcl5*, and exerted pro-proliferative effects on hepatocytes through activation of the AKT/GSK-3β/p27 signaling pathway by CXC/CXCR2 complexes. This study identifies a novel lncRNA with protective effects in acute liver injury and demonstrates that the binding of *Mir122hg*-C/EBPα promotes hepatocyte proliferation via upregulation of CXC chemokine and activation of AKT signaling.

## Liver injury: Protective role for long non-coding RNA

A long non-coding RNA promotes the growth of new healthy cells after liver injury, according to researchers in China. Some long non-coding RNAs incorporate micro-RNA sequences and are recent targets of interest to elucidate their biological roles. By analyzing liver injury datasets, Zhenjun Yu at Tianjin Medical University and co-workers found that a long non-coding RNA called Mir122hg is enriched in liver tissues found in both mice and humans. Using mouse models, the team observed that Mir122hg expression levels dropped immediately after an induced liver injury, but rose significantly when the liver was recovering. Further analyses showed that Mir122hg promotes the gene transcription of important signaling proteins known as chemokines and activates signalling pathways that encourage new healthy liver cells to proliferate. These molecular mechanisms could be exploited in new therapies.

## Introduction

Liver injury is a common pathological basis for the development and progression of many liver diseases and can be induced by various pathogens, including viruses, bacteria, alcohol, drugs, metabolism, and trauma, manifested as inflammatory cell infiltration, cytokine release, and cell death^1^. Severe acute attack resulting in massive hepatocyte (HC) death and shortages of regenerated HCs can lead to acute liver failure, multiple organ failure, and even death^2^. The prognosis of acute liver injury and liver failure is closely related to the degree of cell death and regeneration of residual HCs^3^.

Long noncoding RNAs (lncRNAs) are a class of RNAs >200 nt with no protein-coding potential. Compared to messenger RNAs (mRNAs), lncRNAs are less conserved in species but have cell line-, tissue- or development-specific expression properties^4^. Growing evidence shows that many lncRNAs are aberrantly altered in liver disease and can regulate cellular metabolism, inflammatory response, autophagy, proliferation and apoptosis by direct or indirect regulation of key protein-coding genes^5^. For example, lncRNA-*H19*, which has been reported to have significant regulatory roles in embryonic growth, tumorigenesis, and cell differentiation, can also promote hepatic steatosis through the expression of MLXIPL, a transcription factor that induces lipogenesis and is involved in the development of hepatic lipid metabolism disorders^6^. In studies of acute liver injury, deletion of TUG1 protected against lipopolysaccharide (LPS)-induced hepatocyte inflammation by regulating *miR-140*/TNF^7^. In a fulminant liver failure mouse model induced by D-galactosamine (Dgal)/LPS, overexpressed lncRNA-*NEAT1* resulted in elevated apoptosis and impaired hepatocyte proliferation^8^.

Some lncRNAs are generated from genomic loci that contain microRNAs (miRNAs), and such lncRNAs with miRNA sequences in their genes are also called miRNA-host-gene-derived lncRNAs (lnc-MIRHGs). Approximately 17.5% of miRNAs are produced from lnc-MIRHGs, which are usually thought to be precursor genes of miRNAs or nonfunctional byproducts of miRNA processing^9^. However, recent studies have identified many lnc-MIRHGs that play independent roles in a variety of key biological processes^10^. For example, *MIR100HG* produces spliced and stable lncRNAs that are significantly upregulated in the G1 phase of the cell cycle; it serves as a binding platform for the interaction between HuR and target mRNAs, regulating cell proliferation regardless of the level of mi*R100*^9^.

In this study, we identified the mouse *miR122* host-gene-derived lncRNA (*Mir122hg*), which was further verified to be homologous to human *MIR122HG*. In CCl_4_-treated mice, overexpression of *Mir122hg* attenuated liver injury by promoting hepatocyte proliferation; consistently, exacerbated liver injury was found when *Mir122hg* was silenced. *miR122* levels were unaffected by overexpression or silencing of *Mir122hg*. The possible regulatory mechanism of Mir122hg was its binding and inhibition of the transcription factor CCAAT enhancer-binding protein alpha (C/EBPα), promoting the transcription of downstream chemokines and the activation of protein kinase B (AKT) signaling, which might provide new molecular mechanisms for biomedical and therapeutic applications in liver injury.

## Materials and methods

### RNA-seq data and gene enrichment analysis

Previously published RNA-seq datasets related to liver injury, namely, the paracetamol (APAP)-induced liver injury dataset (GSE111828), partial hepatectomy dataset (GSE125007), and bacterial infection-induced liver injury dataset (GSE122741) (NCBI Gene Expression Omnibus database, GEO), were analyzed using molecular biology methods. Sequence Read Archive files were uncompressed to complete fastqc quality testing, and the Spliced Transcripts Alignment to a Reference software^11^ was used for accurate alignment to obtain the read counts of each gene. R software with the edgeR package was used to analyze the raw gene count data. The differentially expressed genes (DEGs) were screened under the condition of |log2FoldChange | >2, adj. *p* value <0.05, and the DEGs in all three datasets displayed an overlap region in the Venn diagram. Genes closely associated with the target gene were screened under the conditions of |correlation coefficient R | > 0.5 and *p* value <0.05, and Gene Ontology analysis (GO) and Kyoto Encyclopedia of Genes and Genomes pathway analysis (KEGG) were performed by R software with the clusterprofiler package.

### Animals

All 8-week-old male Balb/c mice were purchased from the Institute of Experimental Animal Science, Chinese Academy of Sciences (Beijing, China). All animals were housed in a pathogen-free animal house at Tianjin Medical University on a 12 h light/12 h dark cycle. This study was conducted in strict accordance with approved guidelines and was approved by the Animal Care and Use Committee of Tianjin Medical University.

### Acute liver injury induced by carbon tetrachloride (CCl_4_) and D-gal/LPS

Mice were injected intraperitoneally with CCl_4_ (1 mg/kg) for the acute liver injury model, and different groups of mice were sacrificed at 6 h, 12 h, 24 h, 48 h, 96 h and 8 d after injection. Mice were injected intraperitoneally with Dgal (700 mg/kg)/LPS (100 µg/kg) for the fulminant liver failure model, and different groups of mice were sacrificed at 2 h, 4 h and 6 h after injection. Liver tissues and venous blood were taken for analysis.

### Rapid amplification of cDNA ends (RACE)

RACE experiments were performed with the Smart RACE cDNA Amplification Kit (Clontech, Palo Alto, CA, USA) according to the manufacturer’s instructions, as described previously^12^. The 5′ and 3′ specific primers for *Mir122hg* are shown in Supplementary Table 1. PCR amplification, linearization of the vector pRACE plasmid, cloning and sequencing were performed.

### Nuclear-cytoplasmic fractionation

Cytoplasmic and nuclear RNA extractions from primary HCs and AML12 (mouse immortalized hepatocyte cell) were performed with the PARIS™ Kit Am1921 (Invitrogen, Grand Island, NY, USA) according to the manufacturer’s instructions as described previously^12^.

### Treatment with adeno-associated virus 8 (AAV8) in vivo

Mice were injected with control AAV8-TBG (Addgene, Beijing Zhongyuan, Ltd. Beijing, China) or overexpression AAV8-Mir122hg via the tail vein (1 × 10^11^ viral particles/mouse) for 14 d. Control AAV8-sh-Control (Addgene, Beijing Zhongyuan, Ltd. Beijing, China) or gene silencing AAV8-sh-Mir122hg were injected via the tail vein (1 × 10^11^ viral particles/mouse) for 14 d. Primers for adeno-associated viral plasmid construction are shown in Supplementary Table 2.

### Culture and processing of primary HCs and hepatocyte cell lines

Primary HCs were isolated from mice as previously described^12^ and were cultured in Dulbecco’s modified Eagle’s medium (DMEM, Invitrogen, Camarillo, CA) supplemented with 10% fetal bovine serum (FBS, Gibco, Gaithersburg, MD, USA), 100 units/ml penicillin and 100 μg/ml streptomycin. AML12 and L02 (human immortalized hepatocytes) and HepG2 and Huh7 (human hepatoma cells) were purchased from the Shanghai Cell Bank of the Chinese Academy of Sciences, and the base media were DMEM-F12 (AML12) and Roswell Park Memorial Institute-1640 (Invitrogen, Camarillo, CA) (L02) and DMEM (HepG2 and Huh7) with the same FBS and antibiotics as above. In addition, AML12 cells were cultured with 1× insulin-transferrin-sodium selenite media supplement (Sigma‒Aldrich, St. Louis, MO, USA). Primary HCs and AML12 cells were treated in vitro with tumor necrosis factor alpha (TNFα, 10 ng/ml) (Sigma‒Aldrich, St. Louis, MO, USA) for 12–24 h to induce cell injury. The AKT signaling pathway-specific blocker MK2206 (10 ng/ml) (Med Chem Express, Danvers, USA) was used in primary HCs for 24 h, or the CXC receptor 2 (CXCR2) antagonist SB225002 (10 ng/ml) (Med Chem Express, Danvers, USA) was used in primary HCs for 48 h in the signaling-blockade experiments.

### Treatment with lentivirus (LV) or small interfering RNA (si) in vitro

For gene overexpression, cultured cells were infected with LV-Mir122hg or LV-Control for 48 h (1 × 10^9^ viral particles/well). Primers for LV plasmid construction are shown in Supplementary Table 2. For gene silencing, cultured cells were treated with si-Mir122hg, si-Cebpa, si-MIR122HG, or si-Negative Control (si-NC) (GenePharma, Shanghai, China) transfected by Lipofectamine Max (Invitrogen, Carlsbad, CA) for 36 h. The siRNA sequences are shown in Supplementary Table 3.

### Serum enzyme assay

Serum levels of alanine aminotransferase (ALT), aspartate aminotransferase (AST), and lactate dehydrogenase (LDH) were measured using a commercial test kit (Nanjing Jiancheng Corp., Nanjing, China) according to the manufacturer’s protocol.

### Standard polymerase chain reaction (PCR) and quantitative real-time PCR (qPCR)

Total RNA was extracted using TRIzol reagent (Takara, Dalian, China). RNA was treated with DNase I (Takara, Dalian, China) to remove potential chromosomal DNA contamination prior to cDNA synthesis. First-strand cDNA was synthesized using random primers and AMV Reverse Transcriptase (Thermo Fisher Scientific, Basingstoke, UK), and miRNA first-strand cDNA was synthesized using Tailing Reactions (Sangon Biotech, Shanghai, China). Standard PCRs were performed using Taqase (CoWin Biosciences, Beijing, China). The target PCR products were compared by agarose gel electrophoresis. For qPCR, all reactions were performed according to the manufacturer’s instructions with SYBR Green master mix (Takara, Dalian, China). The primer sequences used for PCR are shown in Supplementary Table 5.

### Western blot (WB) analysis

WB analysis was performed as described previously^12^. The antibodies against PCNA(Cell Signaling, 13110), HMGB1(Cell Signaling, 6893), LC3B(Cell Signaling, 12741), p62(Cell Signaling, 5114), Becline(Cell Signaling, 3495), BAX(abcam, ab32503), BCL2(Proteintech, 12789-1-AP), Cas8(abcam, ab25901), PARP1(Proteintech, 13371-1-AP), AKT(Cell Signaling, 9272), p-AKT(ser473) (Cell Signaling, 4060), p-AKT(thr308) (Cell Signaling, 13038), p-PTEN(ser380)(Cell Signaling, 9551), p-GSK-3β(ser9) (Cell Signaling, 5558), p-c-Raf(ser259) (Cell Signaling, 9421), mTOR(Cell Signaling, 2983), p-mTOR(ser2448) (Cell Signaling, 5536), p27(abcam, ab32034), p-p27(s10)(abcam, ab62364), CDK2(abcam, ab32147), CDK4(Santa Cruz, sc-23896), CyclinB1(abcam, ab181593), CyclinD1(abcam, ab134175), Cyclin E(Santa Cruz, sc-377100), NEDD4 L(Santa Cruz, sc-514954), C/EBPα(Cell Signaling, 8178) and GAPDH(Cell Signaling, 5174) were diluted in TBS containing 5% milk and 0.1% Tween 20. Signals were detected using a chemiluminescence system (Millipore, Bedford, MA, USA).

### Histology and immunohistochemistry (IHC)

Hematoxylin-eosin staining (HE) and IHC analysis were performed as described previously^12^. The antibodies against PCNA (Cell Signaling, 13110), Ki67 (Abcam, ab16667), and HMGB1 (Cell Signaling, 6893) were diluted in phosphate buffered saline (PBS). The reaction products were visualized using diaminobenzidine and monitored by microscopy.

### Confocal microscopy

Primary HCs and AML12 cells were replated on polylysine-precoated glass coverslips. The cells were treated with 4% paraformaldehyde overnight at 4 °C, permeabilized in 1% Triton X-100 for 0–60 min, and blocked with 10% goat serum for 30 min at room temperature. p-AKT (Ser473) (Cell Signaling, 4060) and p27 (Abcam, ab32034) were incubated overnight at 4 °C, and unrelated isotype rabbit IgG (Millipore, PP64B) was used as a negative control. Goat anti-rabbit IgG (Invitrogen, Alexa Fluor 594) was incubated for 1 h at room temperature in the dark, and the nuclei were stained with DAPI (5 µg/ml). The cells were finally washed with PBS and mounted with antifade mounting medium (P0126, Beyotime, Shanghai, China). All immunofluorescence was then visualized by a confocal microscope (LSM 700).

### RNA‒protein pulldown and mass spectrometry

The target linear DNA template was obtained by PCR amplification, agarose gel electrophoresis, and gel recovery with *Mir122hg* overexpression plasmid and primers containing the T7 promoter sequence (Supplementary Table 4) according to the manufacturer’s instructions, followed by application of the TranscriptAid T7 High Yield Transcription Kit (Thermo Fisher Scientific, Basingstoke, UK) for transcription in vitro to obtain the sense and anti-sense RNA of *Mir122hg*. The Pierce^TM^ RNA 3′ END Desthiobiotinylation Kit (Thermo Fisher Scientific, Basingstoke, UK) was used to perform desthiobiotinylation ligation to the 3′ ends of the sense and antisense RNA. The Pierce^TM^ Magnetic RNA‒Protein Pull-Down Kit (Thermo Fisher Scientific, Basingstoke, UK) was then used to perform the RNA‒Protein pulldown experiments. Briefly, 50 pmol biotin-labeled RNA was incubated with 50 µl magnetic beads at room temperature with mixing for 30 min, and the supernatant was removed on a magnetic separator. Nuclear protein lysate from primary HCs, for which nuclear-cytoplasmic protein fractionation was performed with a PARIS™ Kit Am1921, was added and incubated at 4 °C with mixing for 60 min. The target RNA-pulldown proteins were obtained after washing and elution, followed by electrophoresis with polyacrylamide gels and silver staining according to the manufacturer’s instructions (Silver-stain Kit, Beyotime Biotechnology, Shanghai, China). Gel bands of interest were subjected to in-gel digestion as described previously^13^. Then, dehydration in acetonitrile and drying in a SpeedVac (Thermo Fisher Scientific, Basingstoke, UK), trypsin digestion with 50 mM ammonium bicarbonate (Promega Biotech Co., Ltd. China) and desalting with a μ-C18 Ziptip were performed.

Mass spectrum: The digested samples were injected into a Nano-LC system (EASY-nLC 1000, Thermo Fisher Scientific, Basingstoke, UK). Each sample was separated by a C18 column at a flow rate of 300 nL/min. The HPLC eluate was electrosprayed directly onto an Orbitrap Q-Exactive mass spectrometer (Thermo Fisher Scientific, Basingstoke, UK). For the MS1 survey scan, the automatic gain control target was 1e6 with a resolution of 70,000. MS2 spectra were acquired at a resolution of 17,500. MS/MS data were retrieved using Proteome Discoverer software, and the overall false discovery rate of peptides was less than 1%. Proteins with a score <2 were removed.

### RNA binding protein immunoprecipitation assay (RIP)

RIP was performed using the Magna RIP RNA-Binding Protein Immunoprecipitation Kit (Millipore, Bedford, MA, USA) according to the manufacturer’s instructions. Hepatocyte suspensions were isolated from mouse livers and washed twice with precooled PBS. Complete RIP lysis buffer with the same volume of cell pellets was added, mixed gently, incubated on ice for 5 min, transferred to –80 °C, and centrifuged at 4 °C after one freeze‒thaw cycle. Ten microliters of supernatant was transferred to a new EP tube as input, and another 100 µl of supernatant was transferred to the antibody (C/EBPα, Proteintech, 18311-1-AP, 5 µg, and negative control IgG, 5 µg) complex with 900 µl of RIP buffer and incubated overnight at 4 °C with mixing. Afterward, the supernatant was removed on a magnetic separator and washed 6 times repeatedly, followed by elution to purify the RNA for detection.

### Chromatin immunoprecipitation (ChIP)

ChIP was performed using the ChIP Assay Kit (Beyotime Biotechnology, Shanghai, China) according to the manufacturer’s instructions. Briefly, primary HCs were treated with 1% formaldehyde for chromatin cross-linking and terminated with glycine. The cells were washed with PBS, followed by resuspension in 200 µl of SDS lysis buffer and phenylmethylsulfonyl fluoride. Samples were treated on ice with fifteen 10-s pulses for ultrasonic shearing at a power setting of 30% and centrifuged at 14000 × *g* for 10 min. The supernatant was transferred to a new EP tube. Then, 20 µl of supernatant was transferred as input, and the remaining supernatant was added to 1800 µl ChIP Dilution Buffer and incubated with primary antibody (C/EBPα, Proteintech, 18311-1-AP, 5 µg, and negative control IgG, 5 µg) overnight at 4 °C. Then, 60 µl of Protein A/G Agarose was added and incubated with mixing at 4 °C for 1 h. After multiple washes, DNA was eluted and recovered with phenol‒chloroform methods. Primers for ChIP experiments can be found in Supplementary Table 6.

### Human liver tissues

Liver tissues from patients with liver failure (*n* = 6) and healthy liver tissues from patients with hepatic hemangioma (*n* = 6) were obtained from the Liver Transplant Department of Tianjin First Central Hospital. Detailed clinical information of the 12 patients can be found in Supplementary Table 7. This study was approved by the Ethics Committee of Tianjin First Central Hospital, and individual permission was obtained through a standard informed consent procedure. This investigation was in accordance with the Declaration of Helsinki regarding the use of human tissues.

### Statistical analysis

Graphs were plotted using GraphPad Prism software (La Jolla, CA, USA), and WB bands were quantified by ImageJ software (National Institutes of Health, Bethesda, MD, USA). Gene enrichment analysis was performed by R software (version 3.6.3)^14^ (Foundation for Statistical Computing, Vienna, Austria, https://www.R-project.org) with the clusterprofiler package. The data are shown as the mean ± SD of at least triplicate experiments. For statistical analysis of RNA levels, data were calculated using the 2 − ΔΔCt method. Differences between the two groups were analyzed by a two-tailed Student’s *t test*, and *p* < 0.05 was considered statistically significant.

## Results

### Identification and expression profiles of *Mir122hg* in acute liver injury

**Fig. 1:**
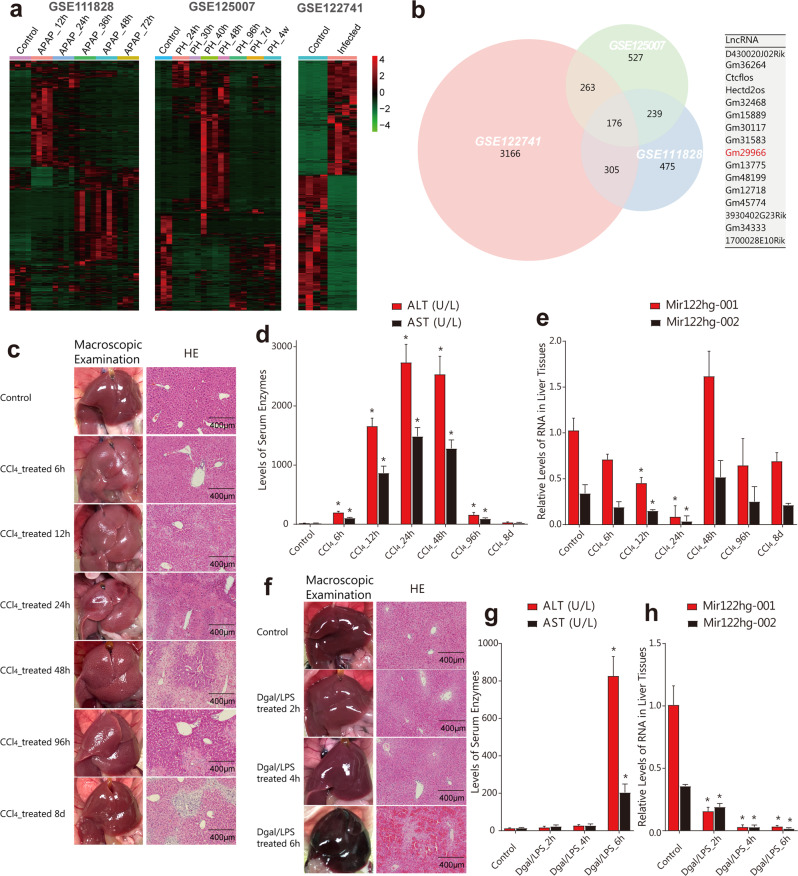
Identification and expression profiles of *Mir122hg* in acute liver injury. **a** Heatmap of DEGs from the APAP-induced, partial hepatectomy, and bacterial infection-induced liver injury RNA-seq datasets from the GEO database, with red representing high expression and green representing low expression. **b** DEGs in three datasets displayed an overlapping region in the Venn diagram, and 176 genes, including 16 lncRNAs, were differentially expressed in all three datasets. **c**–**e** Mice in each group (*n* = 8 per group) were sacrificed at 6 h, 12 h, 24 h, 48 h, 96 h and 8 d after CCl_4_ treatment, and the livers were taken for photographing and HE staining **c**. Venous blood was taken for the detection of serum enzymes **d**. RNA levels of *Mir122hg-001* and *002* in the liver were detected by qPCR **e**. **f**–**h** Mice in each group (*n* = 8 per group) were sacrificed at 2 h, 4 h and 6 h after Dgal/LPS treatment, and the livers were taken for photographing and HE staining **f**; venous blood was taken for the detection of serum enzymes **g**; RNA levels of *Mir122hg-001* and *002* in the liver were detected by qPCR **h**. The data are shown as the mean ± SD of at least triplicate experiments. *p < 0.05 vs. Control.

To investigate the role of lncRNAs in acute liver injury, we analyzed the APAP-induced, partial hepatectomy, and bacterial infection-induced liver injury RNA-seq datasets from the GEO database. The expression heatmap of DEGs in these datasets is shown in Fig. 1a. After intersection of the DEGs, we obtained 176 differentially expressed genes, among which 16 were lncRNAs (Fig. 1b). The 16 lncRNAs were then detected in CCl_4_- and Dgal/LPS-induced mouse models, and *Gm29966* showed a consistent decreasing trend in both models (Supplementary Fig. 1a-d) and the largest enrichment in liver tissues (Supplementary Fig. 1e). Therefore, lncRNA-*Gm29966* was selected as the target gene for subsequent study. Sequence search and genome-wide localization from the Ensembl Genome Browser database (www.ensembl.org/) revealed that the putative *Gm29966* is an intergenic lncRNA with two transcripts, 001 and 002, and transcript 002 covers the full length of *miR122* (Supplementary Fig. 2a). Therefore, we hypothesized that *Gm29966* was the *miR122* host-gene-derived lncRNA and named it *Mir122hg*.

In the CCl_4_-induced mouse model, slight liver injury with partial hepatocyte swelling was already visible in the liver tissue morphology staining at 6 h posttreatment, together with a slight enhancement of serum ALT and AST. Liver injury continued to worsen at 12 h and peaked at 24 h, with high levels of serum ALT, AST, and LDH, and HE staining showed large areas of hepatocyte necrosis with fusion and massive inflammatory cell infiltration. At 48 h, liver damage began to decrease slightly, and at 96 h-8 d, significantly smaller areas of hepatocyte necrosis and a basic return to normal enzyme levels were found (Fig. 1c, d, Supplementary Fig. 2b). In this mouse model from the onset to recovery of acute liver injury, both *Mir122hg-001* and *002* exhibited consistent expression changes in the liver, showing a progressive decrease at 6 h, 12 h and 24 h after CCl_4_ treatment followed by a rapid rebound to above-normal levels at 48 h and a subsequent recovery to normal levels at 96 h and 8 d (Fig. 1e).

In the Dgal/LPS-induced mouse model, no significant changes in morphology or serum enzymes were observed at 2 h and 4 h posttreatment. However, liver damage was sharply aggravated at 6 h, with a dark macroscopic appearance, extensive hepatocyte necrosis and erythrocyte sludge, and significantly elevated serum enzymes (Fig. 1f, g, Supplementary Fig. 2c). In this high-mortality mouse model of fulminant liver failure, *Mir122hg- 001* and *002* exhibited consistent changes in the liver tissues, demonstrating a significant decrease at 2 h after Dgal/LPS treatment and a drop to trough values at 4 h and 6 h (Fig. 1h).

### Properties and distribution of *Mir122hg*

**Fig. 2:**
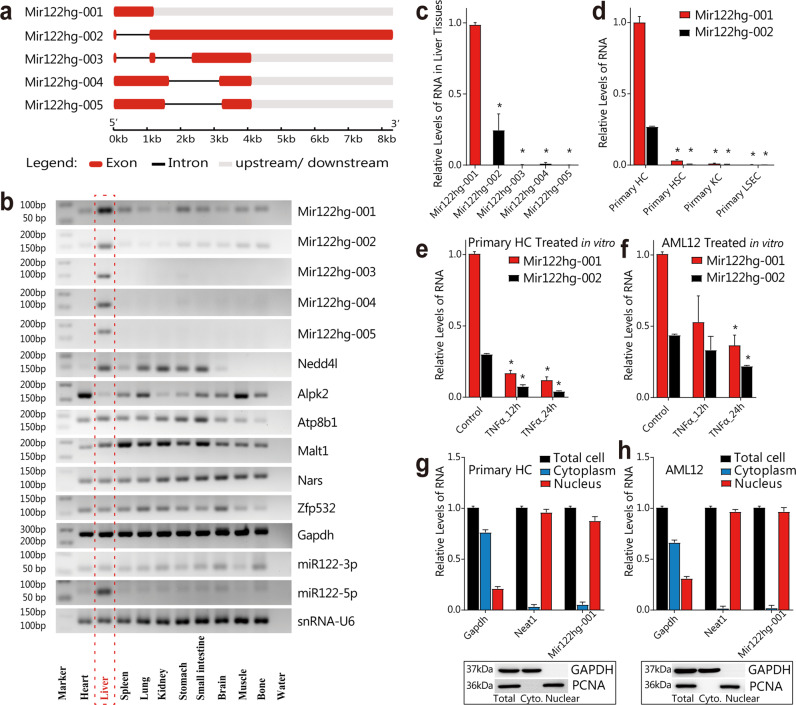
Properties and distribution of *Mir122hg*. **a** Sequences of five transcripts of *Mir122hg* were detected by RACE. **b** Expression of *Mir122hg*, *miR122* and the neighboring genes in 10 organs of mice (*n* = 8) was detected by PCR and agarose gel electrophoresis. **c** Comparison of five transcripts of *Mir122hg* in liver tissues by qPCR. **d** Comparison of *Mir122hg-001* and *002* in primary HCs, HSCs, KCs, and LSECs. **e**, **f** Primary HCs **e** and AML12 **f** were treated with TNFα for 12–24 h in vitro, and the expression levels of *Mir122hg-001* and *002* were measured by qPCR. **g**, **h** Nuclear-cytoplasmic RNA and protein fractionation were performed in primary HCs **g** and AML12 **h**, and the RNA levels of *Gapdh*, *Neat1*, and *Mir122hg-001* and the protein levels of GAPDH and PCNA were measured. The data are shown as the mean ± SD of at least triplicate experiments. *p < 0.05 vs. Control or *Mir122hg-001* or Primary HC.

The *Mir122hg* sequences clarified by RACE were slightly different from the data in Ensembl. The full length of *Mir122hg-001* was 1095 nt, with only one exon and no introns (Supplementary Fig. 2d). In addition, we identified three new transcripts, *Mir122hg-003*, *004*, and *005* (Fig. 2a).

Gene expression in ten organs of normal mice was determined (Fig. 2b). *Mir122hg-001*, *002* and *miR122-5p* were all significantly upregulated in the liver and downregulated in other organs, and transcripts *003*, *004* and *005* were only expressed in the liver. The neighboring gene *Nedd4l* was mainly expressed in the liver, lung, kidney, stomach and small intestine, *Alpk2* in the heart and muscle, *Malt1* in the spleen and lung, and other genes showed no significant differences in different organs (Fig. 2b, Supplementary Fig. 2e, f). Figure 2c shows that *Mir122hg-001* was the most abundant transcript in the liver, and transcripts *003*, *004,* and *005* were all expressed in trace amounts.

Primary HCs, hepatic stellate cells (HSCs), Kupffer cells (KCs), and liver sinusoidal endothelial cells (LSECs) were isolated from mouse livers. Both *Mir122hg-001* and *002* were predominantly expressed in primary HCs (Fig. 2d). TNFα is an injury-induced inflammatory cytokine and is commonly used in studies of hepatocyte injury and death in vitro^15,16^. On the other hand, it has also been reported to promote the expansion of hepatocytes in 3D culture and enable serial passages and long-term culture for more than 6 months^17^. Therefore, primary HCs and AML12 cells were treated with TNFα for the cell injury models in vitro.

Consistent with the results in vivo, *Mir122hg-001* and *002* in primary HCs were significantly decreased at 12 h and 24 h after TNFα treatment (Fig. 2e). Both *Mir122hg-001* and *002* showed low baseline expression in AML12 and were significantly decreased at 24 h, rather than 12 h, after TNFα treatment (Fig. 2f). In addition, *Mir122hg- 001* and *002* were predominantly localized in the nucleus in both primary HCs and AML12, and nuclear localization was not changed in CCl_4_-induced liver injury (Fig. 2g, h and Supplementary Fig. 3a).

As a newly identified gene, we predicted the protein-coding ability of *Mir122hg-001* through the online tools CPC2 and CPAT (http://cpc2.cbi.pku.edu.cn/), and the prediction *p* values were 0.02 and 0.03, respectively. Supported by the characteristic of nuclear localization, it was confirmed as a noncoding gene. Taken together, *Mir122hg-001* and *002*, with significantly higher expression levels for the former, were both mainly located in the hepatocyte nucleus and consistently changed in models of hepatocyte injury in vivo and in vitro. Therefore, our subsequent study mainly focused on the *Mir122hg-001* transcript.

### *Mir122hg* significantly correlates with Ki67 and PCNA in the CCl_4_-induced model

**Fig. 3:**
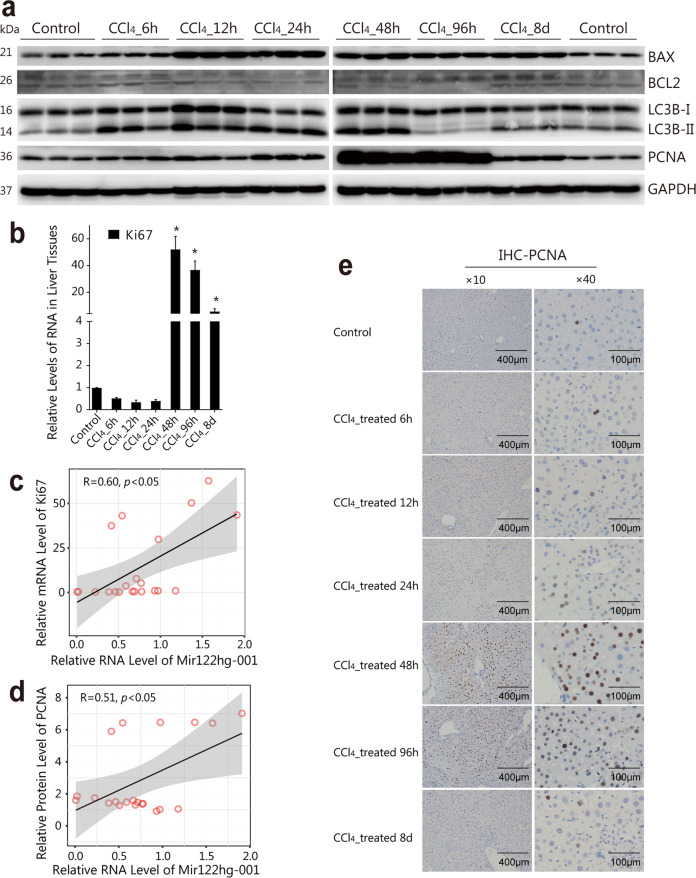
*Mir122hg* significantly correlates with hepatocyte proliferation in the CCl_4_-induced model. **a**–**e** Mice in each group (*n* = 8 per group) were sacrificed at 6 h, 12 h, 24 h, 48 h, 96 h and 8 d after CCl_4_ treatment; protein levels of PCNA, LC3B, BAX and BCL-2 in the liver were detected by WB **a**; mRNA levels of *Ki67* in the liver were measured by qPCR **b**; and PCNA expression was also detected by IHC **e**; mRNA levels of *Ki67* and protein levels of PCNA in each group were analyzed with *Mir122hg-001* by Pearson correlation analysis **c**, **d**. The data are shown as the mean ± SD of at least triplicate experiments. *p < 0.05 vs. Control.

The first finding that drew our attention to the exact role of *Mir122hg* in acute liver injury was the levels of proliferation-related Ki67 and PCNA (Fig. 3a, b). In the CCl_4_-induced model, *Ki67* levels in the liver showed a slight decrease followed by a sharp increase (Fig. 3b), which was in strong agreement with *Mir122hg-001* (correlation R = 0.60, *p* < 0.05) (Fig. 3c). In addition, the protein levels, rather than the mRNA levels, of PCNA were consistent with the trend of *Ki67* (Fig. 3a and Supplementary Fig. 3b, c), and the correlation R value with *Mir122hg-001* was 0.51 (*p* < 0.05) (Fig. 3d). Consistently, IHC analysis of PCNA further showed that hepatocyte proliferation in the CCl_4_-induced model was mild at 24 h, obvious at 48 h and 96 h, and largely normal at 8 d (Fig. 3e). In addition, we also observed a significant change in autophagy-related LC3B-II/I, apoptosis-related BAX, and BCL2, suggesting upregulated hepatocyte autophagy and apoptosis in the CCl_4_-induced model (Fig. 3a).

In the Dgal/LPS-induced model, no obvious change was found in PCNA and Ki67, and the levels of LC3B II/I and BAX were somewhat reduced at 6 h (Supplementary Fig. 3f–i), suggesting a lack of cell proliferation and inhibition of autophagy and apoptosis in this model. In addition, the changes in *miR122* expression in acute liver injury were quite different from those in *Mir122hg*. In the CCl_4_-treated group, the expression of *miR122-3p/5p* was significantly decreased at 48 h and returned to normal at 8 d (Supplementary Fig. 3d, e). In the Dgal/LPS-treated group, the expression levels of *miR122-3p/5p* were both significantly increased at 4 h and 6 h (Supplementary Fig. 3j, k). In conclusion, the CCl_4_-induced model comprehensively reflected the process of liver injury and recovery, and changes in *Mir122hg* were associated with hepatocyte proliferation.

### Attenuation of CCl_4_-induced liver injury in cells overexpressing *Mir122hg* and aggravation of silencing via the direct regulation of hepatocyte proliferation in vivo and in vitro

**Fig. 4:**
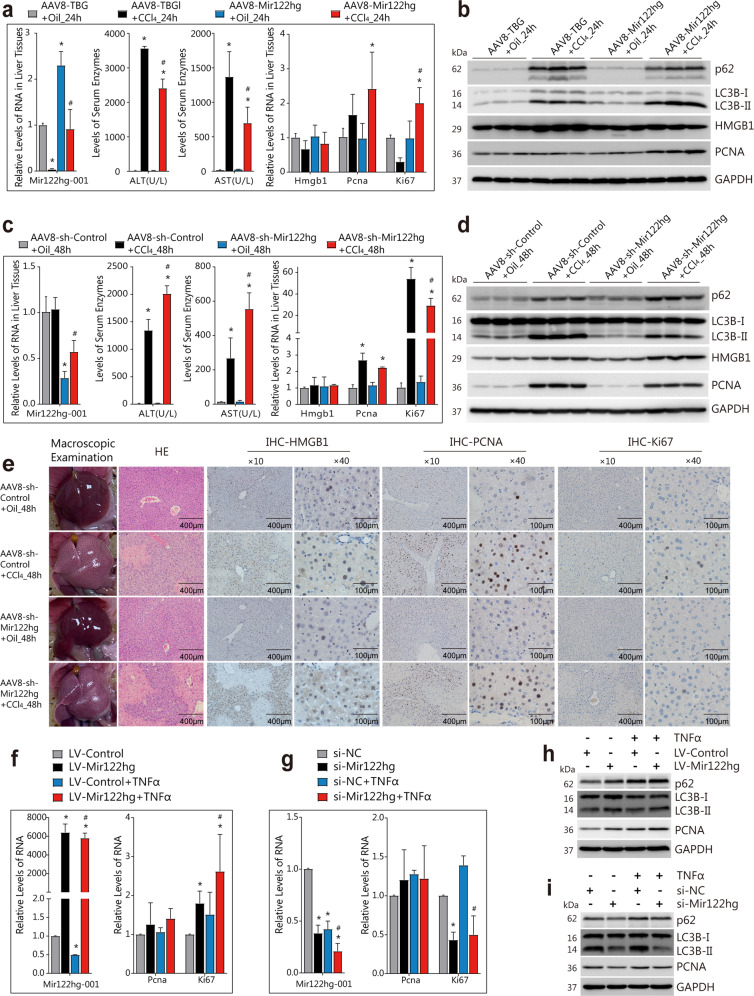
Attenuated CCl_4_-induced liver injury in overexpressed *Mir122hg* and aggravated in the silencing experiments via the direct regulation of hepatocyte proliferation in vivo and in vitro. **a**–**e** For the overexpression experiment in vivo, mice were injected with AAV8-Mir122hg or control AAV8-TBG for 14 d, followed by CCl_4_ or oil treatment for 24 h (*n* = 8 per group); for the silencing experiment in vivo, mice were injected by tail vein with AAV8-sh-Mir122hg or AAV8-sh-control for 14 d, followed by CCl_4_ or oil treatment for 48 h (*n* = 8 per group); RNA levels of *Mir122hg-001*, *Hmgb1*, *Pcna*, *Ki67* in liver and serum ALT, AST of venous blood were detected **a**, **c**; protein levels of PCNA, HMGB1, LC3B, p62 in liver were detected by WB **b**, **d**; liver macroscopic pictures, HE staining, and IHC detection of HMGB1, PCNA, Ki67 were performed, respectively **e**. **f**–**i** For the overexpression experiment in vitro, primary HCs were treated with lentivirus LV-Mir122hg or LV-Control for 48 h, followed by TNFα treatment for 24 h; for the silencing experiment in vitro, primary HCs were treated with si-Mir122hg or control si-NC for 36 h, followed by TNFα treatment for 24 h; the RNA levels of *Mir122hg-001*, *Pcna*, and *Ki67* were measured by qPCR **f**, **g**, and the protein levels of PCNA, LC3B, and p62 were detected by WB **h**, **i**. The data are shown as the mean ± SD of at least triplicate experiments. *p < 0.05 vs. Control, #*p* < 0.05 vs. AAV8-TBG + CCl_4__24 h or AAV8-sh-Control+CCl_4__48 h or LV-Control+TNFα or si-NC + TNFα.

To explore the function of *Mir122hg*, AAV8 was used for overexpression or silencing experiments in the CCl_4_-induced model. Overexpression of *Mir122hg-001* was confirmed by qPCR (Fig. 4a). The upregulation of serum ALT, AST, LDH, protein levels of HMGB1 and liver necrosis at 24 h after CCl_4_ treatment was significantly attenuated in the overexpression group, indicating that overexpressed *Mir122hg* attenuated CCl_4_-induced liver injury (Fig. 4a, b, Supplementary Fig. 4a, c). Both *Mir122hg-001* and *002* were downregulated significantly in the silencing experiment in vivo (Fig. 4c, Supplementary Fig. 4d), and the upregulation of serum enzymes, HMGB1 and liver necrosis at 48 h after CCl_4_ treatment were significantly aggravated, suggesting that silencing *Mir122hg* aggravated CCl_4_-induced liver injury (Fig. 4c–e, Supplementary Fig. 4d).

The mRNA levels of the inflammatory factors *Il-6*, *Il-10*, and *Mcp1* were elevated, except for *Tnfα* and *Il-1β*, at 24 h after CCl_4_ treatment, and inconsistent changes were found in the overexpression group (Supplementary Fig. 4a). Only *Tnfα* and *Mcp1* were significantly elevated at 48 h after CCl_4_ treatment, and no significant change was found in the silencing group (Supplementary Fig. 4d). Therefore, the role of *Mir122hg* in the transcription of these inflammatory factors could not be determined.

As previously described, *Mir122hg* is closely associated with hepatocyte proliferation. The upregulated protein levels of PCNA, autophagy-related LC3BII/I and autophagic substrate SQSTM1 (p62) at 24 h and 48 h after CCl_4_ treatment, including elevated Ki67 at 48 h, which represented increased hepatocyte proliferation and autophagy, were further promoted by overexpressed *Mir122hg* and inhibited by silenced *Mir122hg* in vivo (Fig. 4a–e, Supplementary Fig. 4c).

For further validation in vitro, lentivirus-mediated overexpression and siRNA were used in primary HCs, and the RNA levels of *Mir122hg-001* were confirmed by qPCR (Fig. 4f, g, Supplementary Fig. 4f, h). Overexpression of *Mir122hg* upregulated the mRNA levels of *Ki67* and the protein levels of PCNA, LC3BII/I, and p62, while silencing *Mir122hg* downregulated the above indicators (Fig. 4f–i). Moreover, changes in Ki67, PCNA, LC3B, and p62 regulated by *Mir122hg* were stable in TNFα-induced hepatocyte injury, further suggesting its direct regulation of hepatocyte proliferation and autophagy (Fig. 4f–i). In addition, *Mir122hg* did not affect the levels of apoptosis-related proteins, such as Cas8, BAX, and BCL2, both in vivo and in vitro, suggesting that it does not regulate apoptosis (Supplementary Fig. 4b, e, g, i).

### *Mir122hg* regulates the cell cycle via the AKT/GSK-3β/p27 signaling pathway in vivo and in vitro

AKT signaling is a central survival pathway in liver injury due to its important regulatory role in liver regeneration; hepatocytes and nonparenchymal cells activate AKT signaling through cytokine interactions, promoting hepatocyte proliferation and inhibiting cell death in response to drug or toxic stimuli^18^. Therefore, the changes in AKT and downstream signaling molecules in the CCl_4_-induced model were further investigated in our study.

**Fig. 5:**
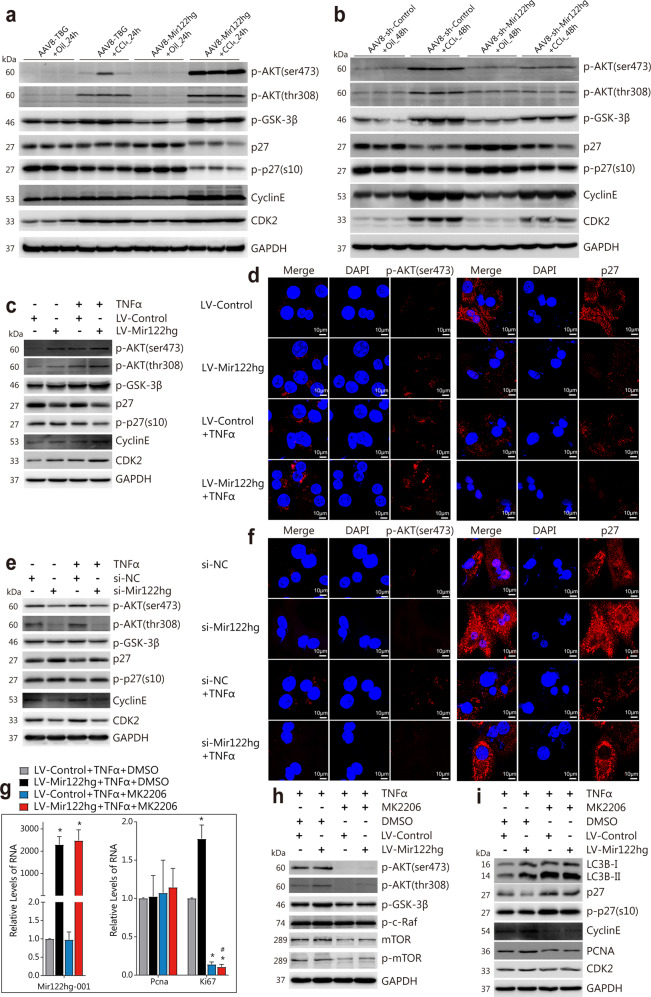
Mir122hg regulates the cell cycle via the AKT/GSK-3β/p27 signaling pathway in vivo and in vitro. **a**, **b** For the overexpression experiment in vivo, mice were injected with AAV8-Mir122hg or control AAV8-TBG for 14 d, followed by CCl_4_ or oil treatment for 24 h (*n* = 8 per group). For the silencing experiment in vivo, mice were injected with AAV8-sh-Mir122hg or AAV8-sh-control for 14 d, followed by CCl_4_ or oil treatment for 48 h (*n* = 8 per group). The levels of AKT signaling-related p-AKT (Ser473), p-AKT (Thr308), p-GSK-3β, cell cycle-inhibitory p27, p-p27, and the cell cycle-related proteins cyclin E and CDK2 in each group were detected by WB **a**, **b**. **c**–**f** For the overexpression in vitro, primary HCs were treated with lentivirus LV-Mir122hg or LV-Control for 48 h, followed by TNFα treatment for 24 h; for the silencing experiment in vitro, primary HCs were treated with si-Mir122hg or control si-NC for 36 h, followed by TNFα treatment for 24 h; cellular p-AKT (ser473), p-AKT (Thr308), p-GSK-3β, p27, p-p27, cyclin E, CDK2 levels were detected by WB **c**, **e**; p-AKT (ser473) and p27 levels were also detected by confocal microscopy **d**, **f**. **g**–**i** Primary HCs were treated with lentivirus LV-Mir122hg or LV-Control for 48 h, followed by treatment with TNFα and AKT specific blocker MK2206 or control DMSO for 24 h; Cellular RNA levels of *Mir122hg-001*, *Pcna*, *Ki67* were measured by qPCR **g**; protein levels of p-AKT(ser473), p-AKT(thr308), p-GSK-3β, p-c-Raf, mTOR, p-mTOR **h**, and downstream proteins LC3B, p27, p-p27, Cyclin E, CDK2, and PCNA were detected by WB **i**. The data are shown as the mean ± SD of at least triplicate experiments. *p < 0.05 vs. Control, #*p* < 0.05 vs. LV-Mir122hg+TNFα + DMSO.

Examination of liver tissues after CCl_4_ treatment showed that the protein levels of phosphorylated p-AKT (ser473), p-AKT (thr308), and p-GSK-3β were increased, the cell cycle inhibitors CDKN1B (p27) and p-p27 were decreased, and the cell cycle-associated proteins CDK2 and Cyclin E were increased, with no significant change in the levels of other AKT-related signaling molecules, such as AKT (pan), mTOR, p-mTOR, p-PTEN, p-c-Raf and cell cycle-related CDK4. AKT signaling and the downstream cell cycle were significantly activated at 24 h and 48 h after CCl_4_ treatment, which were further promoted by overexpressing *Mir122hg* and inhibited by silencing *Mir122hg* in vivo (Fig. 5a, b, Supplementary Fig. 5a, b).

In experiments of primary HCs in vitro, overexpressed *Mir122hg* upregulated the protein levels of p-AKT (Ser473), p-AKT (Thr308), p-GSK-3β, Cyclin E, and CDK2 and downregulated the protein levels of p27 and p-p27, while the exact opposite regulation of the above indicators was found when *Mir122hg* was silenced (Fig. 5c–f). Moreover, changes in p-AKT, p-GSK-3β, Cyclin E, CDK2, p27, and p-p27 regulated by *Mir122hg* were stable in TNFα-induced hepatocyte injury, further suggesting the direct regulation of *Mir122hg* in AKT signaling and the cell cycle (Fig. 5c–f). In addition, no obvious changes in the protein levels of other AKT-related signaling molecules, such as mTOR, p-mTOR, p-PTEN, and p-c-Raf, or the mRNA levels of *Cdk2*, *Cdk4*, *CyclinB1*, *CyclinD1*, and *Cyclin E* were found in *Mir122hg*-overexpressing or -silenced cells in vitro (Supplementary Fig. 5c–f).

For further validation, the AKT signaling pathway-specific blocker MK2206 was used to confirm whether *Mir122hg* regulated the cell cycle via AKT. As shown in Fig. 5h, MK2206 treatment resulted in a significant decrease in the protein levels of p-AKT (Ser473), p-AKT (Thr308), p-GSK-3β, mTOR and p-mTOR and a significant increase in LC3BII/I, suggesting a good blocking effect of AKT signaling (Fig. 5h). Downregulation of p27 and p-p27 and upregulation of Ki67, PCNA, Cyclin E, and CDK2 resulting from overexpression of *Mir122hg* were significantly suppressed after treatment with MK2206, suggesting that the blockade of AKT signaling eliminated the pro-proliferative effect of *Mir122hg* in primary HCs (Fig. 5g–i).

In addition, only overexpression experiments were performed in AML12 cells. Overexpression of *Mir122hg* in AML12 cells did not affect the levels of cell proliferation, autophagy, or the activation of AKT signaling, which was consistent with the results determined by qPCR, WB and confocal microscopy (Supplementary Fig. 6a–d). We speculated that these results were related to the extremely low baseline level of *Mir122hg* in AML12.

### *Mir122hg* regulates CXC chemokine transcription and activates the AKT signaling pathway through CXCR2

Since *Mir122hg* is a nuclear-localized and miRNA host-gene-derived lncRNA, how is it involved in regulating AKT signaling? We first investigated whether it acts as a cis-regulator to regulate the transcription of neighboring genes or as a miRNA precursor. However, the results were negative. In CCl_4_-induced liver injury, correlation analysis showed no significant association between *Mir122hg* and *miR122-3p/5p* (Supplementary Fig. 7a–d). Overexpression or silencing of *Mir122hg* also did not affect the expression levels of *miR122-3p/5p* and its target gene NEDD4L in vivo and in vitro (Supplementary Fig. 7e, f, 8a–d). In addition, no significant change was found in the expression of the neighboring genes in primary HCs treated with overexpressed or silenced *Mir122hg* (Supplementary Fig. 8a, b).

**Fig. 6:**
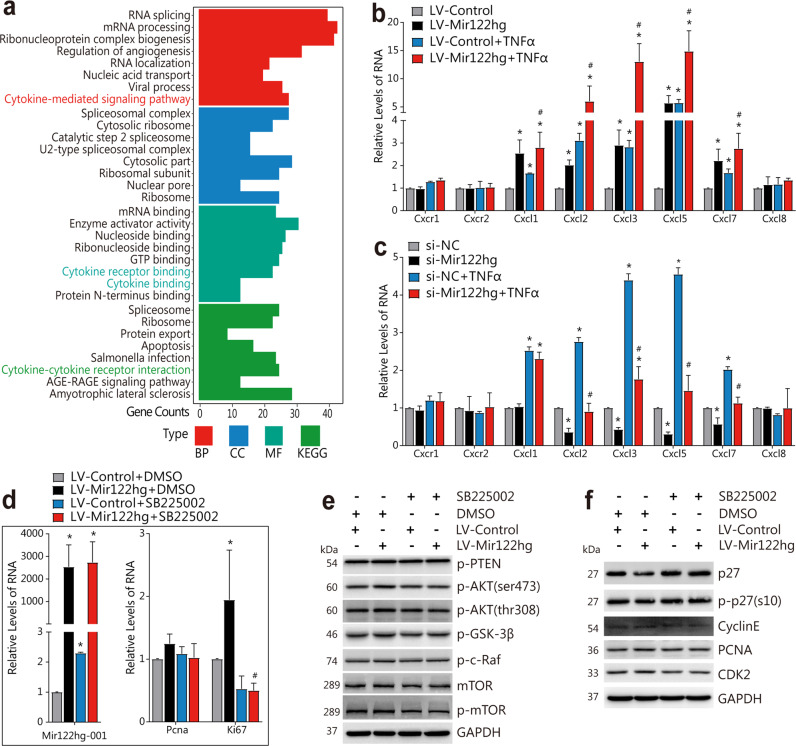
*Mir122hg* regulates CXC chemokine transcription and activates the AKT signaling pathway through CXCR2. **a** Under the condition of |correlation coefficient R | > 0.5 and *p* < 0.05, genes that were closely associated with *Mir122hg* were screened in the three RNA-seq datasets of APAP-, hepatectomy-, and bacterial infection-induced liver injury, and further GO and KEGG analyses were performed by R software with the clusterProfiler package. **b**, **c** For the overexpression in vitro, primary HCs were treated with lentivirus LV-Mir122 hg or LV-Control for 48 h, followed by TNFα treatment for 24 h; for the silencing experiment in vitro, primary HCs were treated with si-Mir122 hg or control si-NC for 36 h, followed by TNFα treatment for 24 h; mRNA levels of Cxcr1, Cxcr2, Cxcl3, Cxcl5, Cxcl7, and Cxcl8 were measured by qPCR. **d**, **f** Primary HCs were treated with lentivirus LV-Mir122hg or LV-Control for 12 h, followed by treatment with CXCR2 specific antagonist SB225002 or control DMSO for 48 h; cellular RNA levels of *Mir122hg-001*, *Pcna*, *Ki67* were measured by qPCR **d**, protein levels of p-AKT(ser473), p-AKT(thr308), p-GSK-3β, p-c-Raf, mTOR, p-mTOR **e**, and p27, p-p27(s10), Cyclin E, CDK2 and PCNA were detected by WB **f**. The data are shown as the mean ± SD of at least triplicate experiments. *p < 0.05 vs. Control, #*p* < 0.05 vs. LV-Control+TNFα or si-NC + TNFα or LV-Mir122hg+ DMSO.

To explore the mechanism of *Mir122hg*, we analyzed the GSE111828, GSE125007, and GSE122741 data again, and 723 genes with significant correlation with *Mir122hg* were identified for further GO and KEGG analysis (Supplementary Table 8). The resultant cytokine-related signaling pathway caught our attention immediately (Fig. 6a).

Chemokines are a class of small molecular proteins belonging to cytokines, including CXC, CC, CX3C and C. The CXC chemokines CXCL1-3 and CXCL5-8 act as ligands for CXCR2, and their binding can affect hepatocyte injury, repair and regeneration, which in turn affects the recovery of the entire liver^19,20^. As shown in Fig. 6b, c, overexpressed *Mir122hg* in primary HCs significantly upregulated the mRNA levels of *Cxcl1*, *Cxcl2*, *Cxcl3*, *Cxcl5*, and *Cxcl7*, and the silencing of *Mir122hg* significantly downregulated the levels of *Cxcl2*, *Cxcl3*, *Cxcl5*, and *Cxcl7*. Moreover, changes in the mRNA levels of these CXC chemokines regulated by overexpressed or silenced *Mir122hg* were stable in TNFα-induced hepatocyte injury, further suggesting the transcriptional regulation of *Mir122hg*.

Further validation was performed in primary HCs with the specific CXCR2 receptor antagonist SB225002, which mildly upregulated the RNA level of *Mir122hg-001*, with no significant changes in AKT signaling-related molecules or cell cycle-related proteins. Therefore, treatment with SB225002 alone did not affect the activation of AKT and the cell cycle but might lead to the compensatory elevation in *Mir122hg* (Fig. 6d–f). On the other hand, upregulation of P-AKT (Ser473), p-AKT (Thr308), p-GSK-3β, Ki67, Cyclin E, PCNA, and CDK2 and downregulation of p27 resulting from overexpression of *Mir122hg* were significantly inhibited by SB225002 (Fig. 6d–f). These results indicated that CXCR2 blockade inhibited the activating effect of *Mir122hg* on AKT signaling and the cell cycle in vitro.

### *Mir122hg* binds C/EBPα and affects its transcriptional repression

**Fig. 7:**
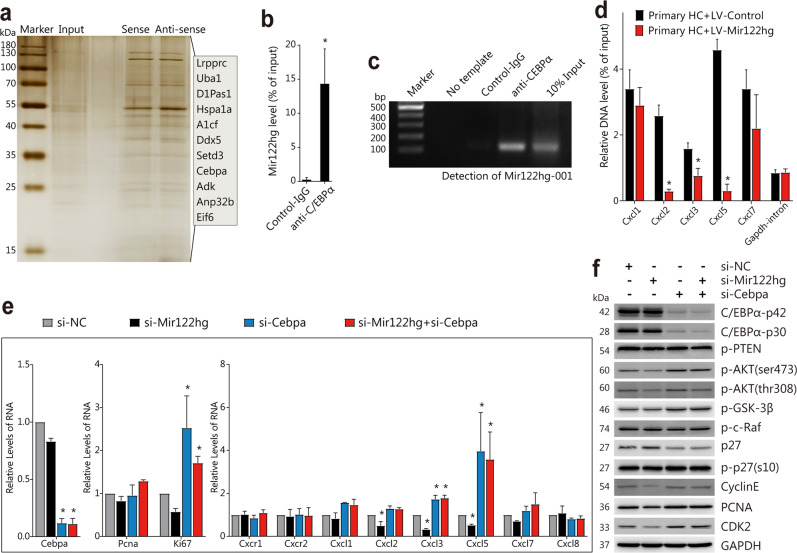
*Mir122hg* binds C/EBPα and affects its transcriptional repression. **a** Sense and antisense strand RNA of *Mir122hg-001* was obtained by in vitro transcription, and RNA‒protein pulldown experiments were performed with biotin magnetic beads; the protein samples obtained were subjected to silver staining, and mass spectrometry revealed 11 possible binding proteins. **b**, **c** qPCR and PCR (including agarose gel electrophoresis) detection of *Mir122hg-001* retrieved by anti-C/EBPα-specific antibody compared with Control IgG in the RIP assay with primary HCs. **d** Primary HCs were infected with LV-Control or LV-Mir122hg, ChIP analyses were performed on the indicated gene promoter regions using an anti-C/EBPα-specific antibody, and enrichment is shown relative to the input. **e**, **f** Primary HCs were treated with si-Mir122hg or si-Cebpa or control si-NC for 36 h. Cellular RNA levels of *Cebpa*, *Pcna*, *Ki67*, *Cxcr1*, *Cxcr2*, *Cxcl1*, *Cxcl2*, *Cxcl3*, *Cxcl5*, *Cxcl7* and *Cxcl8* were measured by qPCR **e**. Protein levels of C/EBPα, p-AKT (ser473), p-AKT (thr308), p-GSK-3β, p-c-Raf, p27, p-p27 (s10), Cyclin E, CDK2 and PCNA were detected by WB **f**. The data are shown as the mean ± SD of at least triplicate experiments. *p < 0.05 vs. Control or si-NC.

To confirm the regulatory mechanism, we labeled the full-length transcript of *Mir122hg-001* with biotin and incubated it for an RNA pull-down assay with nuclear protein lysate from primary HCs. In addition, mass spectrometry was performed to identify the proteins specifically bound to *Mir122hg-001*. Among the 11 possible binding proteins, C/EBPα was selected for further validation because of its cellular localization and functional relevance (Fig. 7a). The specific binding of C/EBPα to *Mir122hg-001* was further confirmed by reverse RIP experiments (Fig. 7b, c).

According to the qPCR and WB results, overexpression or silencing of *Mir122hg* did not affect the mRNA and protein levels of C/EBPα (Supplementary Fig. 8a–d). Previous studies reported the transcriptional repression of chemokines by the transcription factor C/EBPα^21^, and further analysis of the JASPAR database (https://jaspar.genereg.net) identified possible binding sites for C/EBPα to the promoter regions of the downstream CXC chemokine genes *Cxcl1*, *Cxcl2*, *Cxcl3*, *Cxcl5*, and *Cxcl7* (Supplementary Fig. 8e). Overexpression of *Mir122hg* in primary HCs was found to significantly inhibit the binding of C/EBPα to the promoter regions of *Cxcl2*, *Cxcl3*, and *Cxcl5* in the ChIP assay (Fig. 7d). This suggested that Mir122hg might bind and affect the transcriptional repression of C/EBPα, which in turn promoted downstream transcription of CXC chemokines.

Further validation was performed in primary HCs with si-Cebpa to clarify whether silencing C/EBPα in vitro disrupted the *Mir122hg*-C/EBPα complex and affected the function of *Mir122hg*. As shown in Fig. 7e, f, cellular mRNA levels of *Ki67*, *Cxcl3*, and *Cxcl5* were significantly upregulated after C/EBPα silencing in hepatocytes; protein levels of p-AKT (Ser473), p-AKT (Thr308) and downstream p-GSK-3β were upregulated, p27 levels were decreased, Cyclin E and CDK2 levels were significantly upregulated, suggesting that silenced C/EBPα activated the AKT signaling pathway and cell cycle. Moreover, the inhibition of the AKT signaling pathway, cell cycle, and cell proliferation caused by si-Mir122hg in hepatocytes was abrogated by silencing C/EBPα. The above results strongly suggested that Mir122hg exerts its regulatory effects through C/EBPα.

### Identification of the human homologous gene *MIR122HG*

Human *MIR122HG* was reported in only one study^22^. The neighboring genes of *MIR122HG* are the same as those of mouse *Mir122hg* in the Ensembl database; between them, the gene sequence homology is as high as 21%, and the homology region in the genome is where the *miR122* gene is located. Gene expression for each organ in healthy people was obtained from the GTEx database (https://gtexportal.org/), and *MIR122HG* was also found to be significantly enriched in the liver (Supplementary Fig. 9a). *MIR122HG* levels in liver tissues of six patients with liver failure were significantly lower than those in healthy liver tissues (Supplementary Fig. 9b). The baseline levels of *MIR122HG* in L02 and HepG2 cells were much lower than those in Huh7 cells (Supplementary Fig. 9c), which were further selected for silencing experiments in vitro. Silencing *MIR122HG* inhibited cell proliferation, as shown by downregulated mRNA and protein levels of CyclinD1 and protein levels of PCNA (Supplementary Fig. 9d, e). Taken together, these results were sufficient to demonstrate the human-mouse homology of *MIR122HG*.

## Discussion

In this study, we first identified the homologous *Mir122hg* in mice. Consistent with the hepatic specificity of *miR122*, *Mir122hg* showed characteristics of hepatocyte enrichment and nuclear localization and was expressed at low levels in other organs. MiRNAs are 21–22 nt-long ribonucleic acids that often play an important role in regulating biological processes, including the cell cycle, differentiation, development, and immune response, by inhibiting mRNA translation or regulating mRNA stability^23^. As the major miRNA in the liver, *miR122* has been extensively studied and shown to be involved in the regulation of various hepatic processes, including cholesterol and lipid metabolism, mitochondrial function, control of circadian rhythms, and polyploidy regulation^24^. Ashish et al.^22^ first reported human *MIR122HG*, elaborating that most lnc-MIRHG do not use the canonical cleavage and polyadenylation pathway but instead use microprocessor cleavage to terminate transcription; however, the biological function of *MIR122HG* was not investigated in the article.

The lnc-MIRHG loci can produce both miRNAs and lncRNAs, with miRNAs playing a dominant functional role, or both acting synergistically or independently^10^. Lnc-MIRHGs can function as precursors of miRNAs; for example, cancer cells epigenetically regulate the expression of MIRHGs to alter the levels of miRNAs^25^. On the other hand, many lnc-MIRHGs perform functions independently. Similar to other types of lncRNAs, lnc-MIRHGs also function via “competing endogenous RNAs”, “DNA interactors” and “protein interactors” mechanisms^10^. For example, *MIR100HG* plays an oncogenic role in breast cancer by directly interacting with the promoter of p27 to form RNA‒DNA triplex structures, which attenuate the transcription of p27^26^. *MIR31HG* uses its 5′ terminal region to interact with the PAS-B domain of HIF-1α, enhancing the chromatin recruitment of HIF-1α and p300 cofactors to their target gene promoters and promoting the HIF-1 transcriptional network^27^. To explore the possible biological functions of hepatocyte-enriched *Mir122hg*, CCl_4_-induced acute liver injury and Dgal/LPS-induced fulminant liver failure in mice were performed. *Mir122hg* was first sharply decreased at the initial stage of liver injury, but a subsequent increase was only found in the recovered CCl_4_ group, not the severe Dgal/LPS group. *Mir122hg* was significantly correlated with the proliferation-related indices Ki67 and PCNA and was further confirmed to play a protective role in acute liver injury by promoting hepatocyte proliferation in subsequent experiments in vivo and in vitro.

Liver regeneration is an important and necessary process for recovery after liver injury, in which phosphatidylinositol 3-kinase (PI3K)/Akt signaling is the core survival pathway. For example, treatment with the nano-CO donor SMA/CORM2 significantly increased CO levels in the liver and circulatory system and promoted liver regeneration and recovery through the PI3K/AKT/mTOR signaling pathway in APAP-induced liver injury^28^. It was reported that PRDM4 induced cell cycle arrest and downregulated cellular cyclin E, cyclin D1, and CDK4 levels by upregulating p27 and CDKN1A (p21) via inhibition of AKT signaling^29^. Therefore, in this study, we focused on the changes in AKT and downstream signaling molecules. *Mir122hg* promoted hepatocyte proliferation both in vivo and in vitro by activating AKT/GSK-3β signaling, inhibiting p27 expression, and significantly upregulating CDK2, Cyclin E, PCNA, and Ki67 levels during acute liver injury. In addition, our results showed that Mir122hg also promoted hepatocyte autophagy. Paradoxically, activated AKT/mTOR signaling is an important inhibitor of autophagy^30^, so other potential autophagy-related mechanisms are speculated to be involved, which needs to be further explored in the future.

**Fig. 8:**
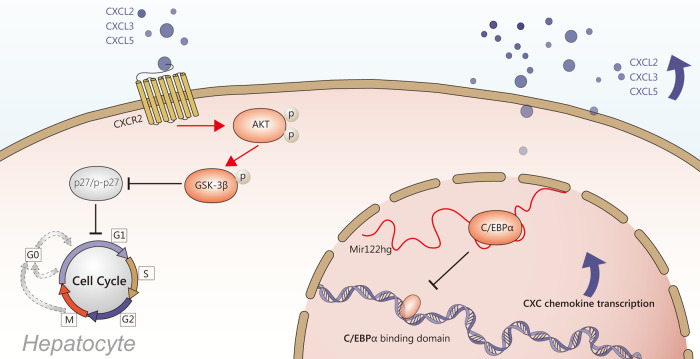
Schematic diagram illustrating the role of lncRNA-Mir122hg in hepatocytes. *Mir122hg* binding to C/EBPα affected its transcriptional repression, promoted the gene transcription of the downstream chemokines *Cxcl2*, *Cxcl3*, and *Cxcl5*, and exerted pro-proliferative effects on hepatocytes through activation of the AKT/GSK-3β/p27 signaling pathway by CXC/CXCR2 complexes.

In general, nuclear lncRNAs play either *cis*-acting or *trans*-acting roles to modulate chromatin, regulate gene transcription or processing, or organize nuclear structures, and cytoplasmic lncRNAs are known to regulate RNA stability, protein translation, and signal transduction^31^. In this study, *Mir122hg* was mainly localized in the nucleus of hepatocytes but did not act through cis-regulatory effects or as a precursor of *miR122*. Combined with the results of gene enrichment analysis, *Mir122hg* promoted gene transcription of downstream CXC chemokines in trans and exerted pro-proliferative effects on hepatocytes through activation of the AKT/GSK-3β/p27 signaling pathway by CXC/CXCR2 complexes (Fig. 8).

The CXC chemokine family plays an important role in liver injury and liver regeneration. It comprises 15 ligands, seven of which (CXCL1-3, CXCL5-8) contain a glutamic acid–leucine–arginine motif, enabling their binding to CXCR2^20^. CXC chemokine levels are upregulated in models of partial hepatectomy; blocking CXC or CXCR2, but not CXCR1, reduces liver regenerative capacity; stimulation of cultured hepatocytes with CXC chemokine results in increased proliferation similar to hepatocyte growth factor induction^19^. It has also been reported that CXCL5 direct treatment of HepG2 cells activates PI3K/AKT and extracellular regulated protein kinase (ERK) signaling with upregulated p-AKT and p-ERK1/2 and promotes cell proliferation, migration and invasion^32^. Liver parenchyma infusion of CXC chemokines successfully promotes stable regeneration of transplanted hepatocytes in the damaged liver^33^. These findings fully illustrate the pro-proliferative effect of CXC chemokines on hepatocytes.

The transcription factor C/EBPα belongs to the C/EBP family and has the highest expression levels in liver and adipose tissue. C/EBPα is an important negative regulator of cell proliferation, the cell cycle and gene expression^34^. In a CCl_4_-induced rat model of liver fibrosis, C/EBPα was significantly decreased in activated HSCs; overexpressed C/EBPα in vitro inhibited HSC activation, extracellular matrix synthesis, αSMA expression, and lipid droplet formation^35^. In another study, aging was found to increase susceptibility to alcoholic liver injury in mice and humans by downregulating the neutrophil Sirtuin1-C/EBPα-Mir223 axis and increasing downstream IL-1β, IL-6, TNFα, and CXCL1 secretion^21^. Consistent with our results, we found transcriptional repression of downstream CXC chemokines by C/EBPα in hepatocytes. Combined *Mir122hg*-C/EBPα inhibits its binding to the promoter regions of the target genes *Cxcl2*, *Cxcl3*, and *Cxcl5*, which in turn promotes transcription. On the other hand, the mechanism regulating the expression of *Mir122hg* in acute liver injury is still not clear and needs to be further studied in follow-up experiments.

In conclusion, our work defines a human-mouse homologous gene and reveals a novel mechanism by which *Mir122hg*-C/EBPα binding promotes CXC chemokine transcription, activates the AKT/GSK-3β/p27 signaling pathway via CXC/CXCR2 complexes, and promotes hepatocyte proliferation. The protective effect of Mir122hg in acute liver injury may become a potential therapeutic target in the future.

## Supplementary information

Supplementary Materials

Supplementary Table 8
