# Supplementary material for: Protective effect of hepatocyte-enriched lncRNA-Mir122hg by promoting hepatocyte proliferation in acute liver injury
Source: Exp Mol Med. 2022 Nov 24;54(11):2022–35. doi: 10.1038/s12276-022-00881-2 (PMC9722683; doi:10.1038/s12276-022-00881-2)
Supplement: Supplementary file 1 — Supplementary Materials [file 12276_2022_881_MOESM1_ESM.pdf]

Supplementary Figures:

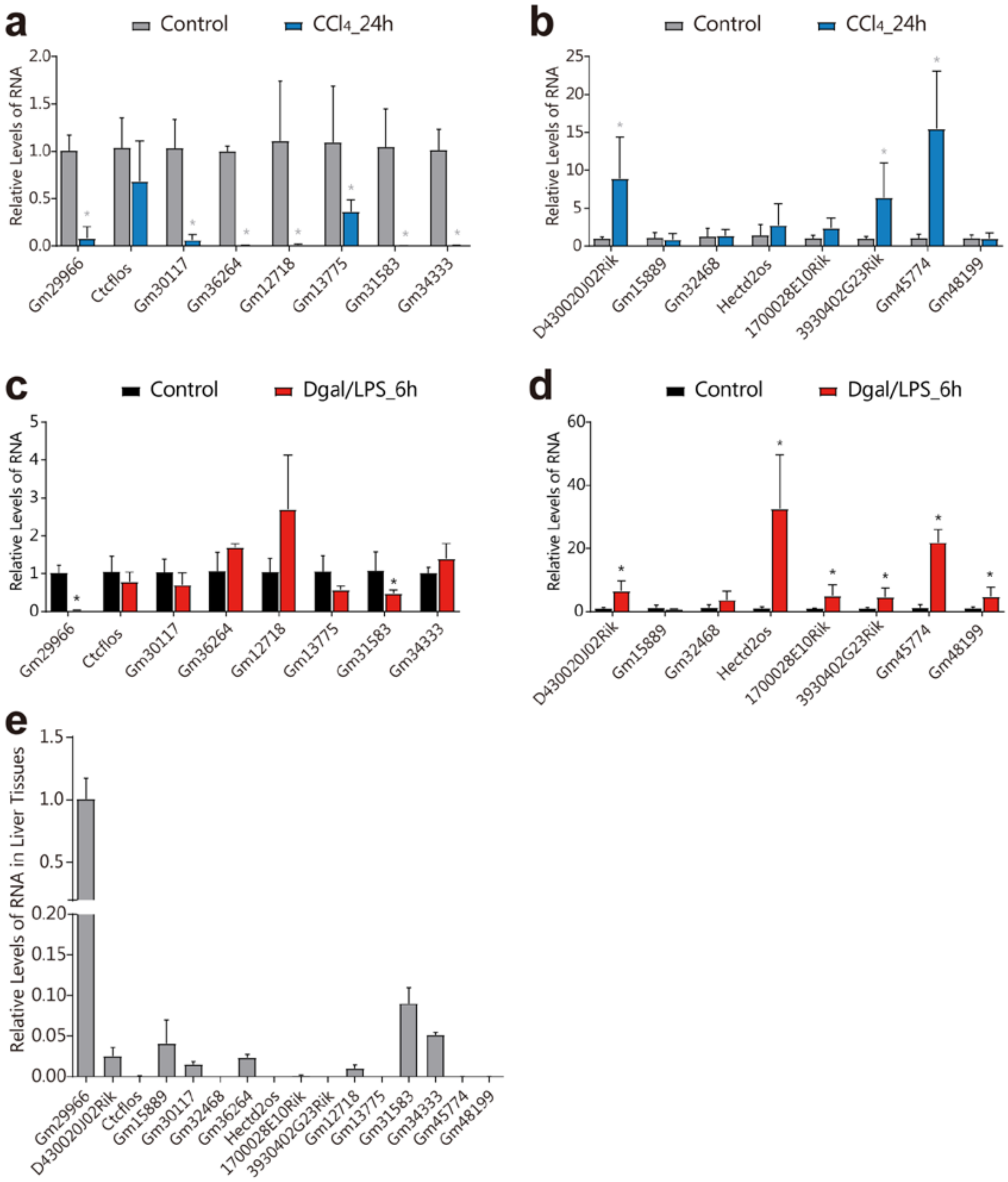

Supplementary Fig. 1: a-d, In the CCl_4-induced model, mice (n=8 per group) were executed at 6h, 12h, 24h, 48h, 96h and 8d after injection of CCl_4; and in the Dgal/LPS-induced model, mice (n=8 per group) were executed at 2h, 4h and 6h after injection of Dgal/LPS; for the pre-experiment, the expression levels of 16 lncRNAs in liver tissues were detected in the control group, the CCl_4-24h group, and the Dgal/LPS-6h group. e, Comparative analysis of 16 lncRNAs expressions in nomal liver tissues were performed. The data were shown as the mean±SD for at least triplicate experiments. *p <0.05 for vs Control.

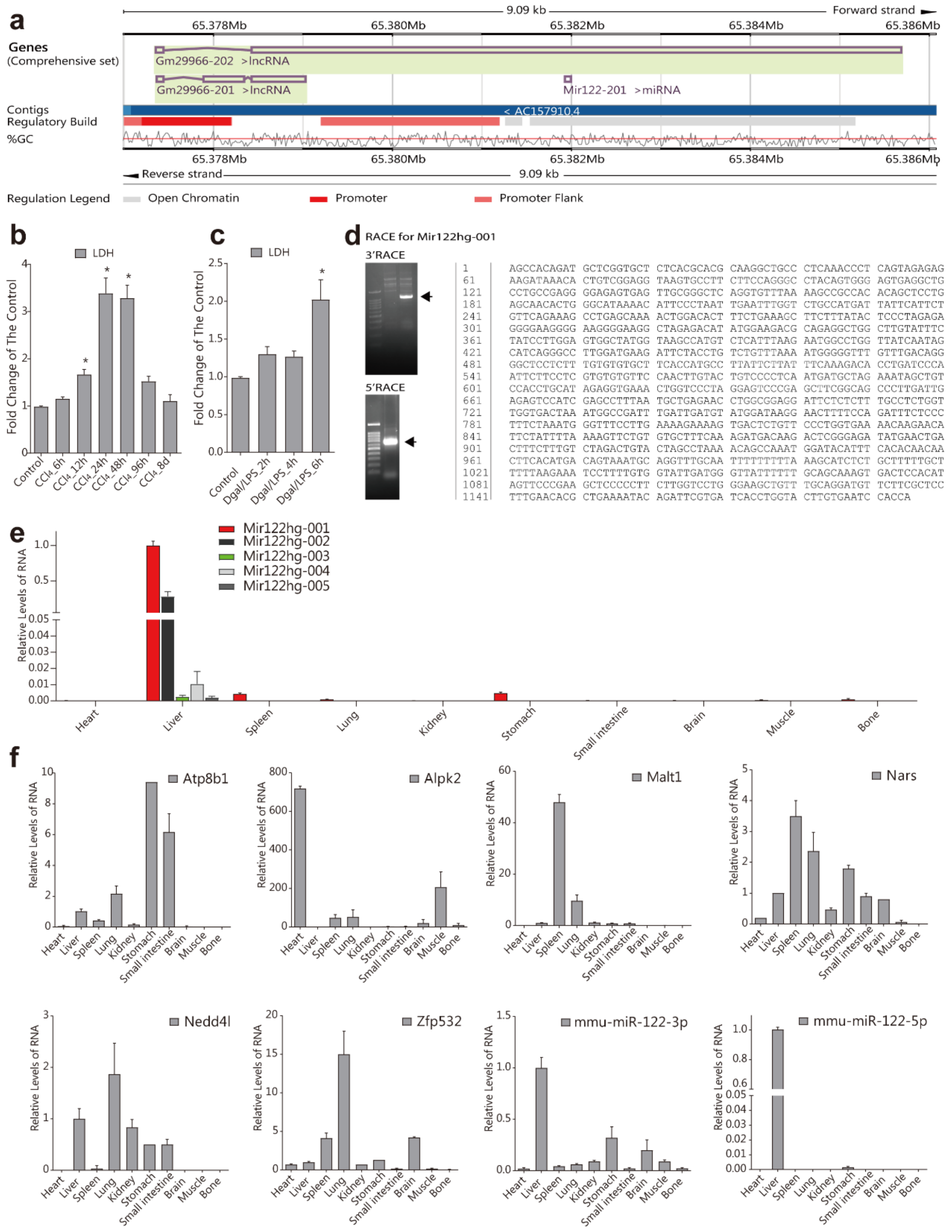

Supplementary Fig. 2: a, Sequence search and genome-wide localization in the Ensembl database revealed that the putative *Gm29966* is an intergenic lncRNA with two transcripts, 001 and 002, and transcript 002 covers the full length of *miR122*. b-c, In the CCl_4-induced model, mice (n=8 per group) were executed at 6h, 12h, 24h, 48h, 96h and 8d after injection of CCl_4; and in the Dgal/LPS-induced model, mice (n=8 per group) were executed at 2h, 4h and 6h after injection of Dgal/LPS, and serum LDH levels were detected, respectively. d, Exact sequences of *Mir122hg* transcripts with or without PolyA tail at the 3' end were identified by 3' RACE, respectively, and the sequences of Mir122hg transcripts at the 5' end was identified by 5' RACE, and the full length of *Mir122hg-001* was obtained after splicing. e-f, Comparative expression of *Mir122hg*, *miR122* and upstream and

downstream neighboring genes in 10 tissues of mice (n=8) were detected by qPCR. The data were shown as the mean±SD for at least triplicate experiments. *p <0.05 for vs Control.

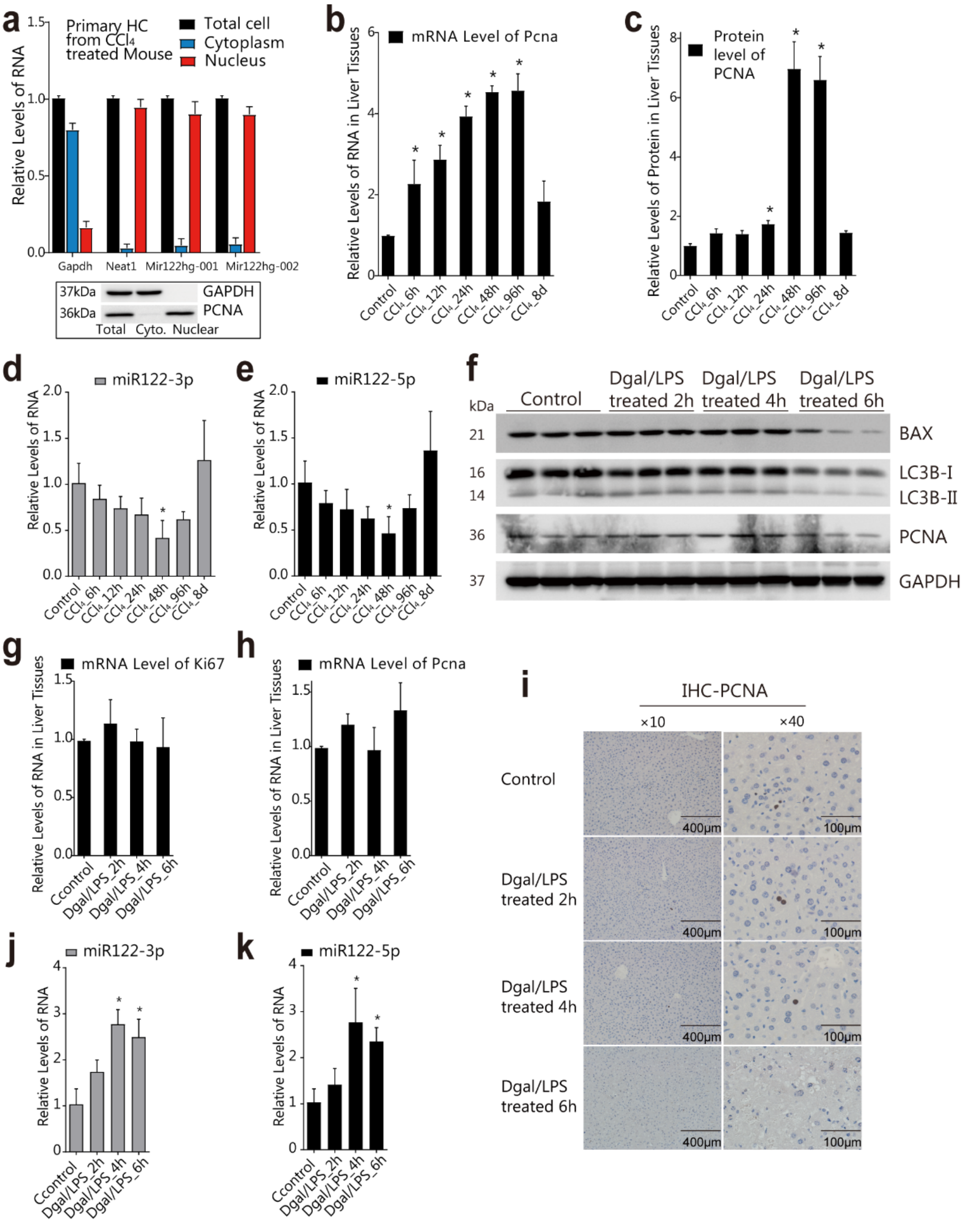

Supplementary Fig. 3: a, Mice were executed at 24h after CCl_4 injection, and primary HCs were extracted for nuclear-cytoplasmic RNA and protein fractionation; the RNA levels of *Gapdh*, *Neat1*, *Mir122hg-001/002* and protein levels of GAPDH and PCNA were detected, respectively. b-e, Mice in each group (n=8 per group) were executed at 6h, 12h, 24h, 48h, 96h and 8d after CCl_4 treatment, respectively; RNA levels of *Pcna*, *miR122-3p*, *miR122-5p*, and protein levels of PCNA in liver were detected, and the WB bands were quantified by ImageJ software, and the relative protein level were calculated based on the PCNA/GAPDH ratio. f-k, Mice in each group (n=8 per group) were executed at 2h, 4h and 6h after Dgal/LPS treatment, respectively; the protein levels of PCNA, LC3B

and BAX in liver were detected by WB (f); the RNA levels of *Ki67*, *Pcna*, *miR122-3p*, and *miR122-5p* in liver were detected by qPCR (g/h/j/k); and PCNA levels were also detected by IHC (i). The data were shown as the mean±SD for at least triplicate experiments. *p <0.05 for vs Control.

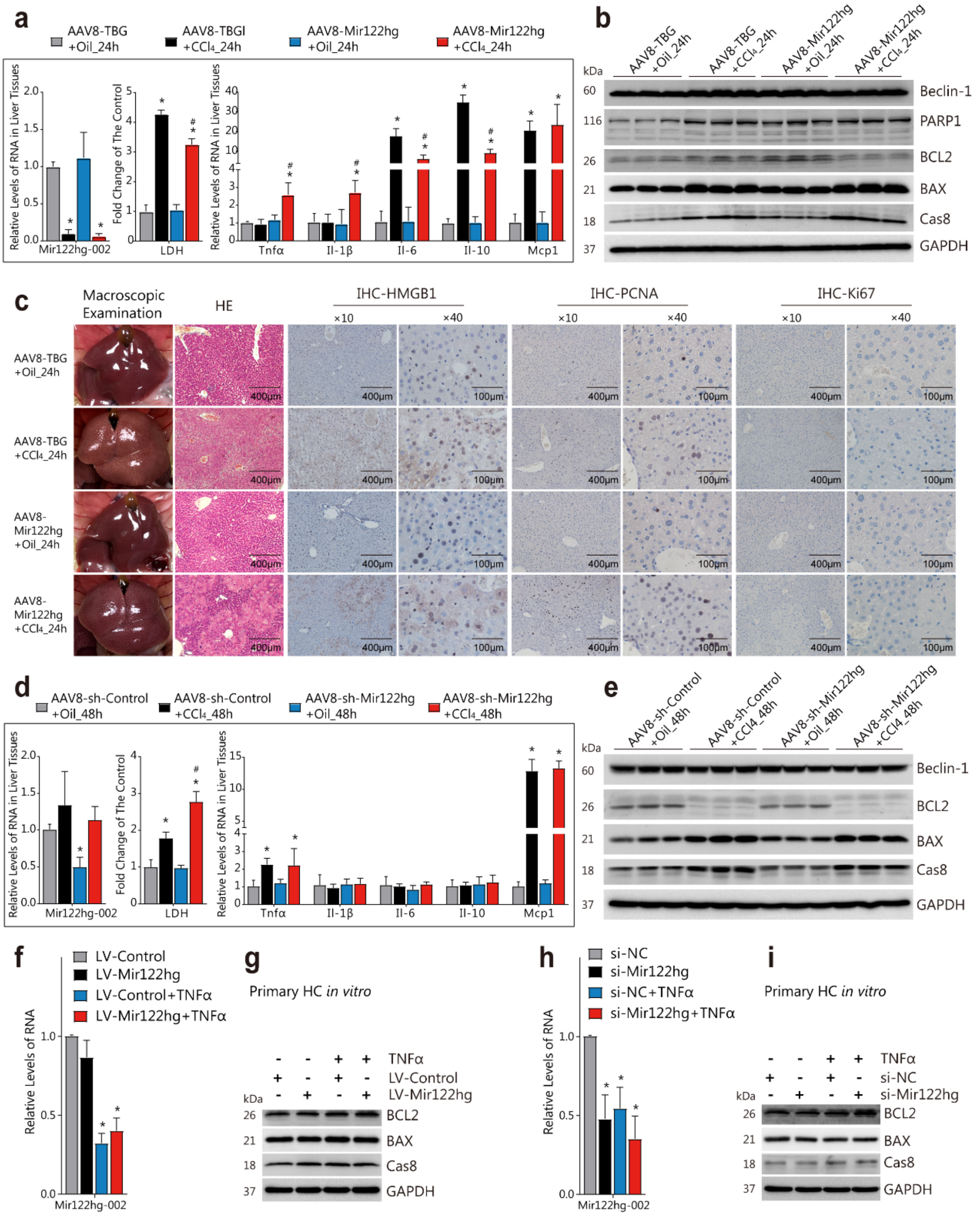

Supplementary Fig. 4: a-e, For the overexpression experiment *in vivo*, mice were injected with AAV8-Mir122hg or control AAV8-TBG for 14d, followed by CCl_4 or Oil treatment for 24h (n=8 per group); and for the silencing experiment *in vivo*, mice were injected by tail vein of AAV8-sh-Mir122hg or AAV8-sh-control for 14d, followed by CCl_4 or Oil treatment for 48h (n=8 per group); the RNA levels of *Mir122hg-002*, *Tnfα*, *Il-1β*, *Il-6*, *Il-10*, *Mcp1* in liver and serum LDH of venous blood were detected (a/d); the levels of autophagy-related Beclin-1, apoptosis-related PARP1, BCL-

2, BAX, Cas8 were detected by WB (b/e); liver macroscopic pictures, HE staining, and IHC detection of HMGB1, PCNA, Ki67 were performed, respectively (c). fi, For the overexpression *in vitro*, primary HCs were treated with lentivirus LV-Mir122hg or LV-Control for 48h, followed by TNFα treatment for 24h; and for the silencing experiment *in vitro*, primary HCs were treated with si-Mir122hg or control si-NC for 36h, followed by TNFα treatment for 24h; the RNA levels of *Mir122hg-002* were detected by qPCR (f/h), and the protein levels of apoptosis-related Cas8, BCL2, BAX in were detected by WB (g/i). The data were shown as the mean±SD for at least triplicate experiments. *p <0.05 for vs Control, #p <0.05 for vs AAV8-TBG+CCl_4_24h or AAV8-sh-Control+CCl_4_48h.

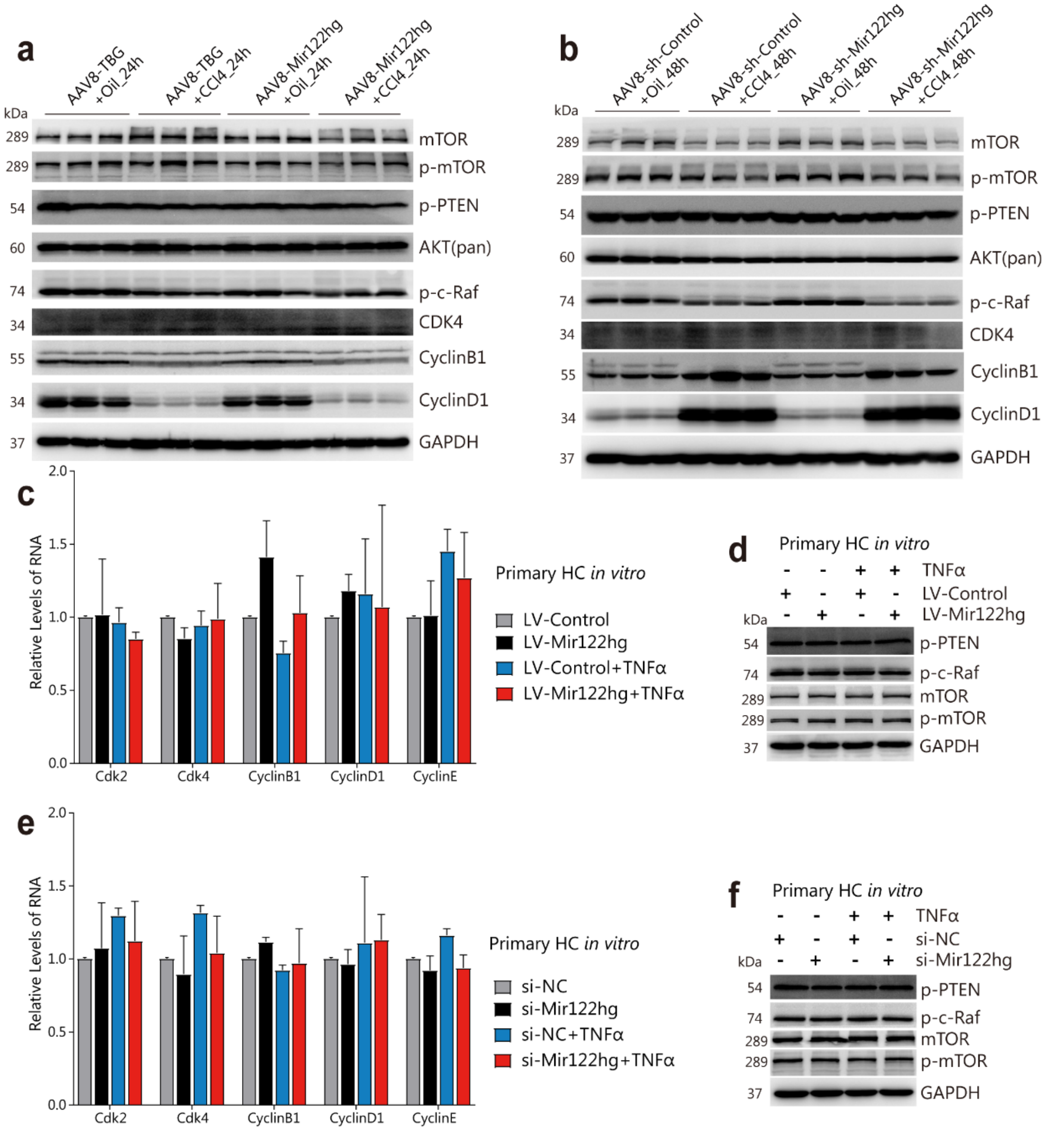

Supplementary Fig. 5: a-b, For the overexpression experiment *in vivo*, mice were injected with AAV8-Mir122hg or control AAV8-TBG for 14d, followed by CCl_4 or Oil treatment for 24h (n=8 per group); and for the silencing experiment *in vivo*, mice were injected by tail vein of AAV8-sh-Mir122hg or AAV8-sh-control for 14d, followed by CCl_4 or Oil treatment for 48h (n=8 per group); the levels of AKT, p-PTEN, p-c-Raf, mTOR, p-mTOR in AKT signaling, and cell-cycle-related CDK4, CyclinB1, CyclinD1 were detected by WB (a/b). c-f, For the overexpression *in vitro*, primary

HCs were treated with lentivirus LV-Mir122hg or LV-Control for 48h, followed by TNFα treatment for 24h; and for the silencing experiment *in vitro*, primary HCs were treated with si-Mir122hg or control si-NC for 36h, followed by TNFα treatment for 24h; the RNA levels of *Cdk2*, *Cdk4*, *CyclinB1*, *CyclinD1*, *CyclinE* were detected by qPCR (c/e), and the protein levels of AKT signaling related p-PTEN, p-c-Raf, mTOR, p-mTOR were detected by WB (d/f). The data were shown as the mean±SD for at least triplicate experiments.

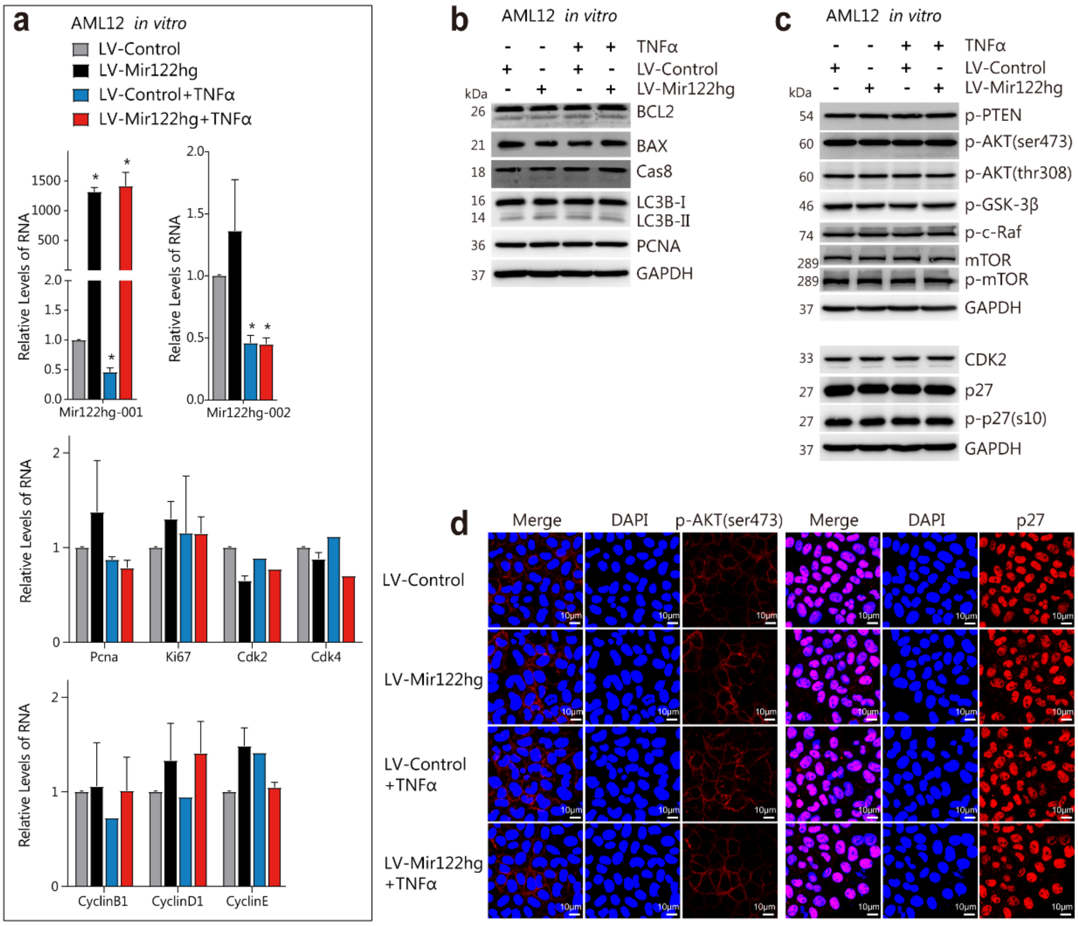

Supplementary Fig. 6: a-d, For the overexpression experiment in AML12, it was treated with lentivirus LV-Mir122hg or LV-Control for 48h, followed by TNFα treatment for 24h; the RNA levels of *Mir122hg-001/002*, *Pcna*, *Ki67*, *Cdk2*, *Cdk4*, *CyclinB1*, *CyclinD1*, *CyclinE* were detected by qPCR (a); protein levels of PCNA, LC3B, Cas8, BAX, BCL-2 (b), and AKT signaling-related p-AKT(ser473), p-AKT(thr308), p-GSK-3β, p-PTEN, p-c-Raf, mTOR, p-mTOR, and downstream p27, p-p27, CDK2 were detected by WB (c); p-AKT(ser473), p27 levels were also detected by confocal (d).

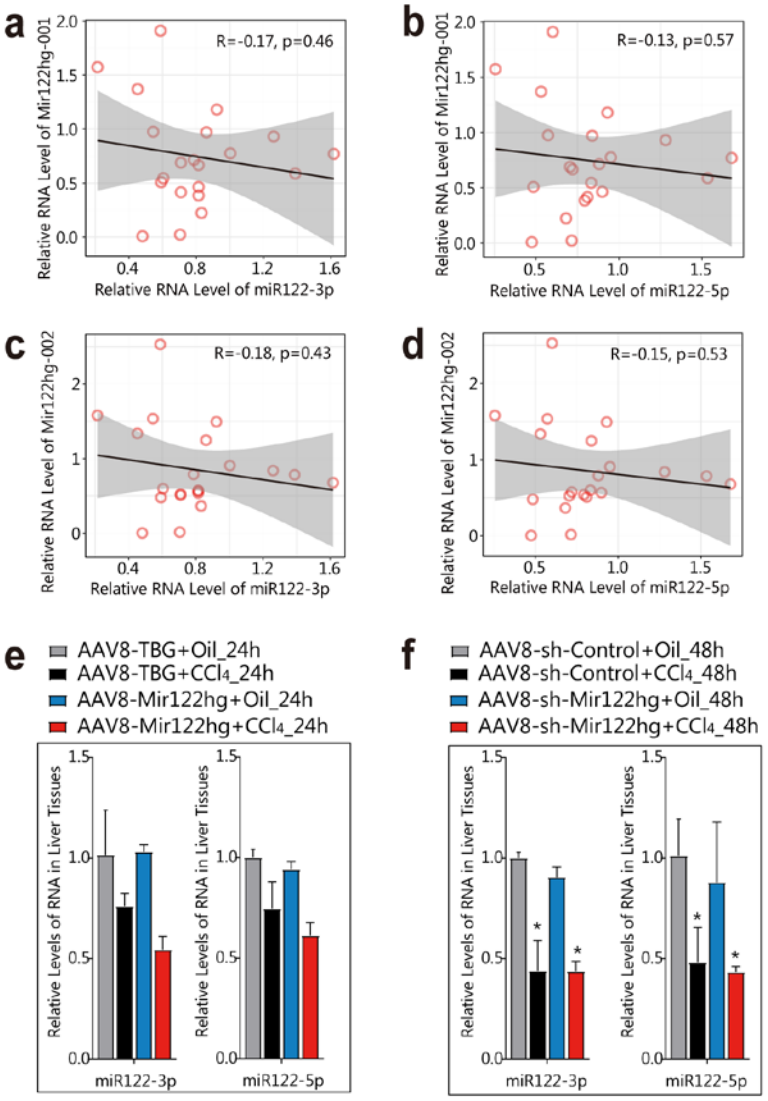

Supplementary Fig. 7: a-d, Mice in each group (n=8 per group) were executed at 6h, 12h, 24h, 48h, 96h and 8d after CCl_4 treatment; correlation analysis between the expression levels of *miR122-3p/5p* and *Mir122hg-001/002* in liver tissues were performed, respectively. e-f, For the overexpression experiment *in vivo*, mice were injected with AAV8-Mir122hg or control AAV8-TBG for 14d, followed by CCl_4 or Oil treatment for 24h (n=8 per group); and for the silencing experiment *in vivo*, mice were injected by tail vein of AAV8-sh-Mir122hg or AAV8-sh-control for 14d, followed by CCl_4 or Oil treatment for 48h (n=8 per group); the RNA levels of *miR122-3p* and *-5p* in liver tissues were detected. The data were shown as the mean±SD for at least triplicate experiments. *p <0.05 for vs Control.

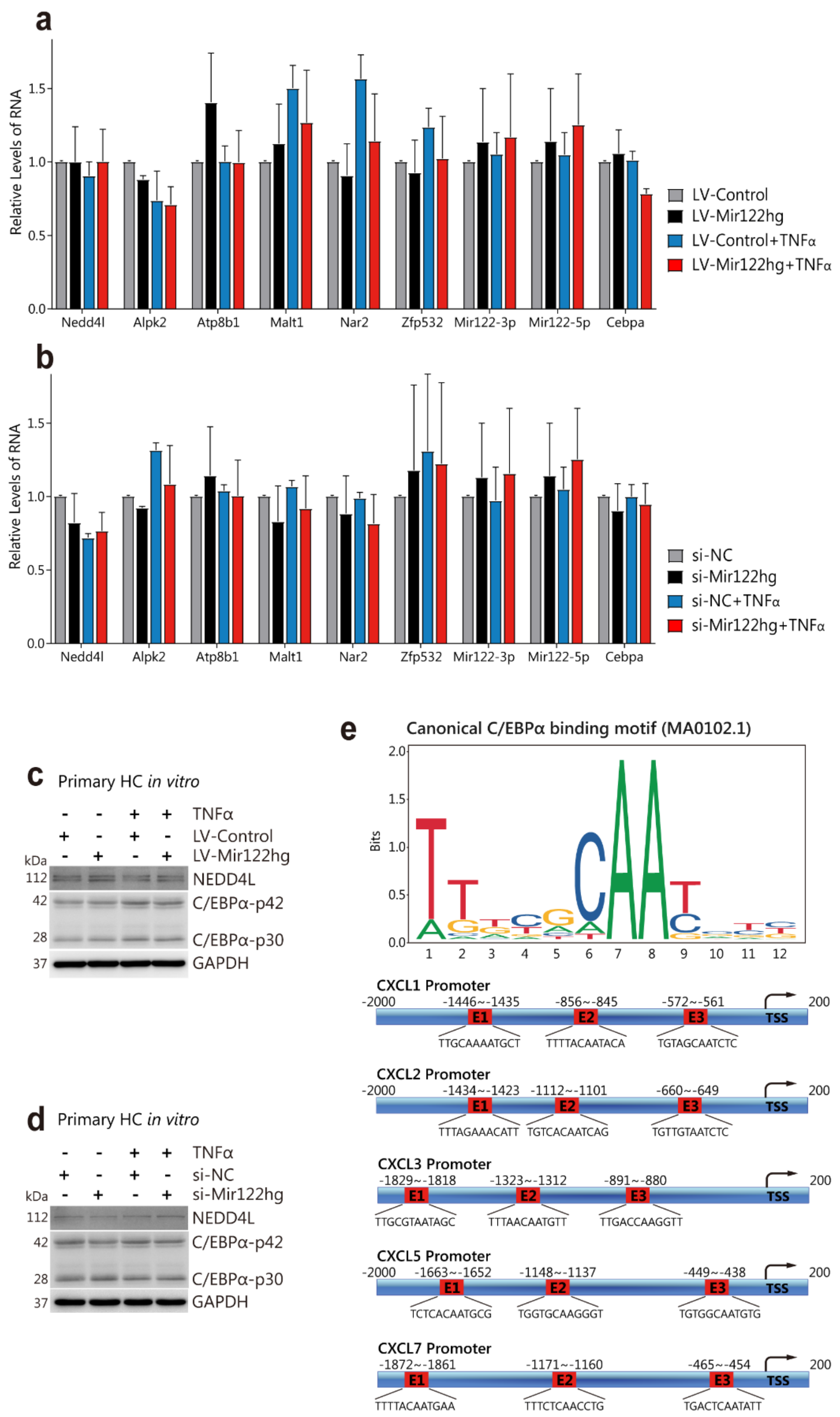

Supplementary Fig. 8: a-d, For the overexpression *in vitro*, primary HCs were treated with lentivirus LV-Mir122hg or LV-Control for 48h, followed by TNFα treatment for 24h; and for the silencing experiment *in vitro*, primary HCs were treated with si-Mir122hg or control si-NC for 36h, followed by TNFα treatment for 24h; the RNA levels of *Nedd4l*, *Alpk2*, *Atp8b1*, *Malt1*, *Nar2*, *Zfp532*, *miR122-3p*, *miR122-5p* and *Cebpa* were detected by qPCR (a/b), and the protein levels of NEDD4L and C/EBPα were detected by WB (c/d). e, The possible binding sites for C/EBPα to the promoter

regions of CXC chemokines *Cxcl1*, *Cxcl2*, *Cxcl3*, *Cxcl5*, and *Cxcl7* were obtained from the JASPAR database. The data were shown as the mean±SD for at least triplicate experiments.

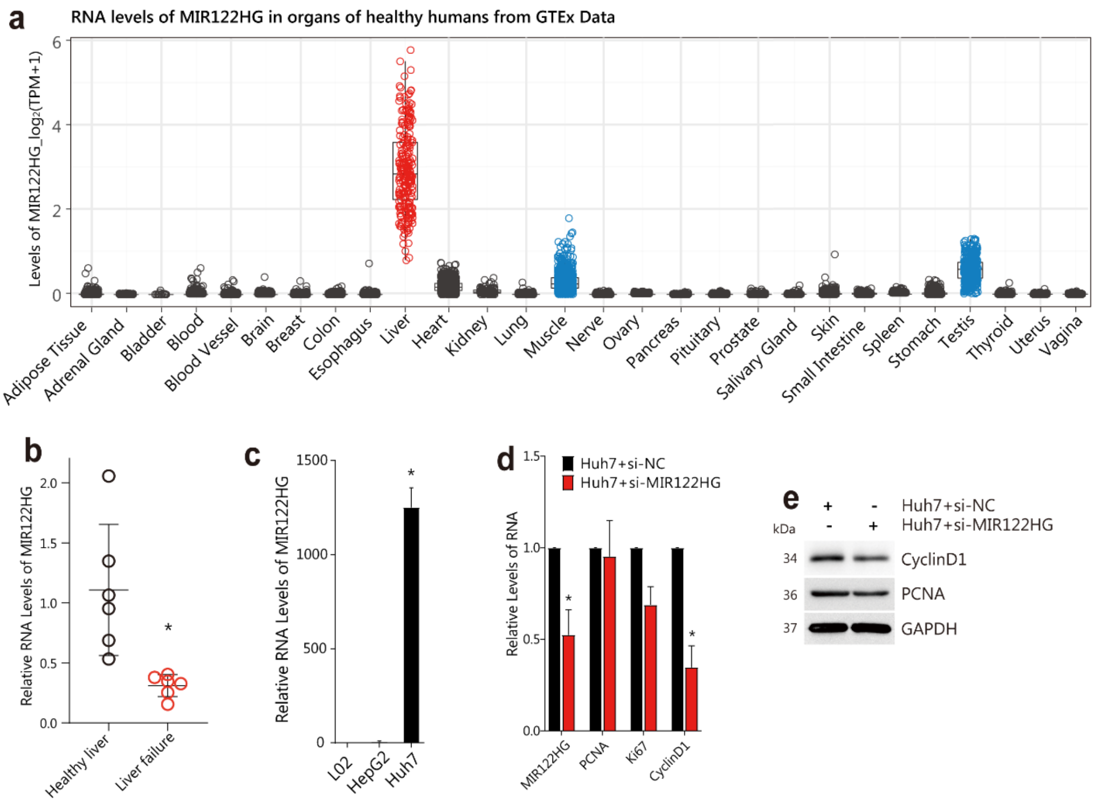

Supplementary Fig. 9: a, RNA-seq data of healthy human from the GTEx database was analyzed to show the levels of MIR122HG in various organs. b, RNA levels of *MIR122HG* in liver tissues from patients with liver failure and healthy liver tissues from patients with hepatic hemangioma were detected by qPCR. c, Cellular RNA levels of *MIR122HG* in L02, HepG2, and Huh7 were detected by qPCR. d-e, For the silencing experiment *in vitro*, Huh7 cells were treated with si-Mir122hg or si-NC for 36h, the RNA levels of *MIR122HG*, *PCNA*, *Ki67*, and *CyclinD1* were detected by qPCR (d), and the protein levels of PCNA, CyclinD1 were detected by WB (e). The data were shown as the mean±SD for at least triplicate experiments. *p <0.05 for vs Control or L02 or Huh7+si-NC.

Supplementary Tables

Supplementary Table1: RACE primers for Mir122hg

Gene Specific Primer (GSP)	Sequence 5' - 3'
Forward GSP	GATTACGCCAAGCTTCGGTGCTCTCACGCACGCAAGGCTG
Reverse GSP	GATTACGCCAAGCTTGGGCTGCCGAAGCTCGGGACTCCTA

Supplementary Table2: Cloning primers for Mir122hg

Plasmid	Name	Sequence 5' - 3'
Lentivirus overexpression plasmid	oe_Forward_Mir122hg-001	CTCTCTCCCCAGGGGGATCCACAGATGCTCGGTGCTCTCA
	oe_Reverse_Mir122hg-001	GACTCGACTCTAGAGGATCCGCAGTCAGAGTGCCTTATTTCC
Adeno associated viral overexpression plasmid	oe_AAV8_Forward_Mir122hg-001	CAAGTAATAAGCTTGGATCCACAGATGCTCGGTGCTCTCA
	oe_AAV8_Reverse_Mir122hg-001	TCCAGAGGTTGATTGGATCCGCAGTCAGAGTGCCTTATTTCC
Adeno associated viral silencing plasmid	sh_AAV8_Forward_Mir122hg-001	GATCCGCCTGGTTATCAATAGCATTTCAAGAGAATGCTATTGATAACCAGGCTTTTTTG
	sh_AAV8_Reverse_Mir122hg-001	AATTCAAAAAAGCCTGGTTATCAATAGCATTCTCTTGAAATGCTATTGATAACCAGGCG

Supplementary Table3: siRNA sequences

Name	Forward 5' - 3'	Reverse 5' - 3'
si-Mir122hg (mouse)	GCCUGGUUAUCAAUAGCAUTT	AUGCUAUUGAUAACCAGGCTT
si-Cebpa (mouse)	GGAACAGCUGAGCCGUGAATT	UUCACGGCUCAGCUGUUCCTT
si-MIR122HG (human)	GAGCUAAGAGACUUCUAGATT	UCUAGAAGUCUCUUAGCUCTT
si-NC (negative control)	UUCUCCGAACGUGUCACGUTT	ACGUGACACGUUCGGAGAATT

Supplementary Table4: Primers for *in vitro* transcription

	Name	Sequence 5' - 3'
sense RNA_ Mir122hg-001	Forward_1 (including T7)	TAATACGACTCACTATAGGGCACAGATGCTCGGTGCTCTC
	Reverse_1	CAGCGCCTAGGAAGATGTGG
antisense RNA_ Mir122hg-001	Forward_2	CACAGATGCTCGGTGCTCTC
	Reverse_2 (including T7)	TAATACGACTCACTATAGGGCAGCGCCTAGGAAGATGTGG

Supplementary Table5: qPCR primers for analysis of transcript levels

Gene symbol	Forward 5' - 3'	Reverse 5 '- 3'
Mouse Mir122hg-001_1	GCCTGGCATTAGACAGGGAG	GGGGCTGTGGACAATTCCAA
Mouse Mir122hg-001_2	CCTGCATAGAGGTGAAACTGGT	GGCTCGATGGACTCTCAATCA
Mouse Mir122hg-001_3	CACTGTCGGAGGTAAGTGC	GTGTTGCTCAGGAGCTGTG
Mouse Mir122hg-002	CTAGGCAATCCGTCCACTCC	GGAAGGCGTCAGTCAGATGT
Mouse Mir122hg-003	CACTGTCGGAGCAAAGTGAC	CAGTGCTCAGATGCATCAGC
Mouse Mir122hg-004	CCAACACCAAAGGTTTTAGG	CTGCAGGAAAAGAGTGTCA
Mouse Mir122hg-005	GAAATGTCTAGAAATCCCATCC	CAGGAAAAGAGTGTCTTATTTCC
Mouse miR122-3p	CGCGAAACGCCATTATCACACTAAAT	GTGCAGGGTCCGAGGTATTC
Mouse miR122-5p	CCTGGAGTGTGACAATGGTGTTTG	GTGCAGGGTCCGAGGTATTC
Mouse snRNA-U6	AGACAATTGATGCGTGCGATC	GCTGCAACTGCACTACCAAC
Mouse Nedd4l	CACGGGTGGTGAGGAATCC	GCCGAGTCCAAGTTGTGGT
Mouse Alpk2	CTCCTCCCCAAAGAACCGAC	CTGCTGGTGGTCGAAGGATT
Mouse Atp8b1	GGATGCAAGCTGTCATGTCC	TGGGCTGAGTAGCCATTGAA
Mouse Malt1	GACAGTCAAGATAGCAAGAAGGC	TGGTCAATTCATACACATCCACC
Mouse Nars	TTCTGGGAACTAATTGGGTTGGC	CCTCGGATCATCATGTGTCTGTT
Mouse Zfp532	TCAGGAAAGTTTACGCCTGCT	GGTCCTTGATCCCGTGCAT
Mouse Neat1	GCTCTGGGACCTTCGTGACTCT	CTGCCTTGGCTTGGAAATGTAA
Mouse Gapdh	GGCATGGACTGTGGTCATGAG	TGCACCACCAACTGCTTAGC
Mouse Hmgb1	GGCGAGCATCCTGGCTTATC	GGCTGCTTGTCATCTGCTG
Mouse Pcna	TTTGAGGCACGCCTGATCC	GGAGACGTGAGACGAGTCCAT
Mouse Ki67	CATCCATCAGCCGGAGTCA	TGTTTCGCAACTTTCGTTTGTG
Mouse Tnfα	CATCTTCTCAAAATTCGAGTGACAA	TGGGAGTAGACAAGGTACAACCC
Mouse Il1-β	GTCGCTCAGGGTCACAAGAA	GTGCTGCCTAATGTCCCCTT
Mouse Il-6	AGTTGCCTTCTTGGGACTGA	TCCACGATTTCCCAGAGAAC
Mouse Il-10	GCTCTTACTGACTGGCATGAG	CGCAGCTCTAGGAGCATGTG
Mouse Mcp1	CCACAACCACCTCAAGCACT	AGGCATCACAGTCCGAGTCA
Mouse Cdk2	CCTGCTTATCAATGCAGAGGG	GTGCTGGGTACACACTAGGTG
Mouse Cdk4	ATGGCTGCCACTCGATATGAA	TCCTCCATTAGGAACTCTCACAC
Mouse CyclinB1	AAGGTGCCTGTGTGTGAACC	GTCAGCCCCATCATCTGCG
Mouse CyclinD1	GCGTACCCTGACACCAATCTC	CTCCTCTTCGCACTTCTGCTC
Mouse CyclinE	GTGGCTCCGACCTTTCAGTC	CACAGTCTTGTCAATCTTGGCA
Mouse Cxcr1	CAAACAATGGCCGAGGCTG	CAGCAAGCTCAGAAGGGAC
Mouse Cxcr2	ATGCCCTCTATTCTGCCAGAT	GTGCTCCGGTTGTATAAGATGAC
Mouse Cxcl1	CTGGGATTCACCTCAAGAACATC	CAGGGTCAAGGCAAGCCTC
Mouse Cxcl2	CCAACCACCAGGCTACAGG	GCGTCACACTCAAGCTCTG
Mouse Cxcl3	GAAAGGAGGAAGCCCCTCAC	ACACATCCAGACACCGTTGG
Mouse Cxcl5	CACTCGCAGTGGAAAGAACG	CGTGGGTGGAGAGAATCAGC
Mouse Cxcl7	ATTGCAACGGAAATCGCCTG	ATGTTGCAGAGGTTGCTTGG
Mouse Cxcl8	GACAGAGATACCGCCACGTT	CAGTGTTCTTGCCTTGGCTC
Mouse Cebpa	CAAGAACAGCAACGAGTACCG	GTCACTGGTCAACTCCAGCAC

Human MIR122HG	GTGAAATGGGTCCAAGCCCT	TCGCTGACCATCCACAAGAG
Human Ki67	GCCTGCTCGACCCTACAGA	GCTTGTCAACTGCGGTTGC
Human PCNA	GGTTACTGAGGGCGAGAAGC	GACCGGCTGAGACTTGCGTA
Human CyclinD1	GTGGCCTCTAAGATGAAGGAGA	GGAAGTGTTCAATGAAATCGTG
Human GAPDH	ACCCAGAAGACTGTGGATGG	TTCAGCTCAGGGATGACCTT

Supplementary Table6: Primers for Mouse ChIP qRT-PCR

Locus	Forward 5' - 3'	Reverse 5 '- 3'
Cxcl1(pro-1485,-1375)	GTAGAGTACCACGTCTCTTCC	GCATTTGCTTCACAGAGCTG
Cxcl1(pro-941,-768)	GCTAGTTCTAGGTAACCCTG	GTCTAACAGCAAAGGGCTAT
Cxcl1(pro-640,-527)	GATGCTTCAGGAACCACACC	CTGTCAAGGTTAGGTCCCTG
Cxcl2(pro-1459,-1376)	CCTTAGCATTTATACAGTGAGT	GAAATGCACCCAAATAACTG
Cxcl2(pro-1138,-1039)	CTAGAACTGAGGGCTACAATAG	GTATACATCCATTCTTGTCCCA
Cxcl2(pro-688,-565)	CCAGAATTTCACAGAGGAAGT	CATATCCTTCTACCCGACTTG
Cxcl3(pro-1888,-1709)	GATCTCTGTAAATCTGAGGC	GTCATCACACTCAGTTTGTC
Cxcl3(pro-1412,-1288)	GCAAAAGTGTACCTTCCAG	GTGTTACAGGCATTACAACA
Cxcl3(pro-942,-829)	CCTTCTTAGCCTTAGTGTCT	GCATCTGAAACTCCTGATTG
Cxcl5(pro-1719,-1620)	CATTTGTTGAGTCAAGTGTG	GTCTAGTTGAAGGAAGTTGG
Cxcl5(pro-1186,-1111)	CAGAACGGATGCTATCTTGC	CTCATCCGTGGGACTTATCT
Cxcl5(pro-474,-334)	CTAGAGAAGCTTGTGAGCAG	CAAACACTATTGCTGACACC
Cxcl7(pro-1977,-1801)	GCTAAGAGACTTTGCTATCC	CTACCATTTGTATAAACTCCTTC
Cxcl7(pro-1219,-1137)	GTGACTCTGTGAATGGGTTG	CAAGCTGGCTTCAAACTCAC
Cxcl7(pro-516,-416)	CAGAGAGAAGCCCATAAACT	CTGCTGGGTATCAGAATTTG
Gapdh intron	ATCCTGTAGGCCAGGTGATG	AGGCTCAAGGGCTTTTAAGG

Supplementary Table7: Clinical information of the 12 patients with hepatic hemangioma and liver failure.

Group	Age(years)	Gender	ALT(U/L)	AST(U/L)	TB(umol/L)	LDH(U/L)	ALB(g/L)
Healthy liver tissues (from patients with hepatic hemangioma)	48	Male	12.1	15.1	13.1	130	40.2
	35	Female	16.2	15.5	12.4	125	41.3
	55	Female	16.9	15.9	7.6	134	38.5
	36	Male	16.1	27.2	5.3	210	39.2
	45	Male	23.3	19.4	8.2	207	40.5
	63	Male	19.4	19.1	7.7	185	41.2
Liver failure tissues (from patients with acute liver failure or acute-on-chronic liver failure)	54	Male	506.1	246.6	369.1	250	30.9
	44	Male	366.5	274.3	12.9	156	47.6
	56	Male	349.8	925.7	162.7	563	27.6
	50	Male	148.3	374.4	201.5	366	36.2
	52	Male	105.3	226.9	185.7	211	30
	34	Female	3649.6	2404.7	67.1	262.5	36.1
